# Metabolic and Transcriptional Analysis of Acid Stress in *Lactococcus lactis*, with a Focus on the Kinetics of Lactic Acid Pools

**DOI:** 10.1371/journal.pone.0068470

**Published:** 2013-07-03

**Authors:** Ana Lúcia Carvalho, David L. Turner, Luís L. Fonseca, Ana Solopova, Teresa Catarino, Oscar P. Kuipers, Eberhard O. Voit, Ana Rute Neves, Helena Santos

**Affiliations:** 1 Instituto de Tecnologia Química e Biológica, Universidade Nova de Lisboa, Oeiras, Portugal; 2 Department of Molecular Genetics, Groningen Biomolecular Sciences and Biotechnology Institute, University of Groningen, Haren, The Netherlands; 3 Integrative BioSystems Institute and the Wallace H. Coulter Department of Biomedical Engineering, Georgia Institute of Technology and Emory University, Atlanta, Georgia, United States of America; 4 Departamento de Química, Faculdade de Ciências e Tecnologia, FCT, Universidade Nova de Lisboa, Caparica, Oeiras, Portugal; Technion-Israel Institute of Technology, Israel

## Abstract

The effect of pH on the glucose metabolism of non-growing cells of *L. lactis* MG1363 was studied by *in vivo* NMR in the range 4.8 to 6.5. Immediate pH effects on glucose transporters and/or enzyme activities were distinguished from transcriptional/translational effects by using cells grown at the optimal pH of 6.5 or pre-adjusted to low pH by growth at 5.1. In cells grown at pH 5.1, glucose metabolism proceeds at a rate 35% higher than in non-adjusted cells at the same pH. Besides the upregulation of stress-related genes (such as *dnaK* and *groEL*), cells adjusted to low pH overexpressed H^+^-ATPase subunits as well as glycolytic genes. At sub-optimal pHs, the total intracellular pool of lactic acid reached approximately 500 mM in cells grown at optimal pH and about 700 mM in cells grown at pH 5.1. These high levels, together with good pH homeostasis (internal pH always above 6), imply intracellular accumulation of the ionized form of lactic acid (lactate anion), and the concomitant export of the equivalent protons. The average number, *n*, of protons exported with each lactate anion was determined directly from the kinetics of accumulation of intra- and extracellular lactic acid as monitored online by ^13^C-NMR. In cells non-adjusted to low pH, *n* varies between 2 and 1 during glucose consumption, suggesting an inhibitory effect of intracellular lactate on proton export. We confirmed that extracellular lactate did not affect the lactate: proton stoichiometry. In adjusted cells, *n* was lower and varied less, indicating a different mix of lactic acid exporters less affected by the high level of intracellular lactate. A qualitative model for pH effects and acid stress adaptation is proposed on the basis of these results.

## Introduction

Bacterial survival depends on the ability to perceive and adapt to fluctuations in the environmental conditions. At the molecular level, the adaptive response is characterized by a readjustment of components at every level of biological complexity, ultimately shaping the cellular physiology to endure the new condition. A stress response generally results from the combination of a general component involving genes related to transcription and protein synthesis, as well as the repair of damage (e.g., chaperones, chaperonins, DNA repair machinery), and a specific component intrinsically dependent on the type of stress imposed (pH, temperature, osmotic, oxidative) [1,2]. With respect to acid stress, neutrophilic organisms have developed a number of mechanisms to withstand low pH conditions [3,4]. Hitherto, the majority of studies on the acid stress response has been limited to gene expression analyses [5], and a lack of information at the metabolic level is still hampering a comprehensive understanding of the stress response at the systemic level, as recognized by a number of recent reports [6].

*Lactococcus lactis* is a mesophilic Gram-positive bacterium of great economic value due to its worldwide application in a variety of dairy fermentations. Despite its contribution to the organoleptic and nutritional properties of the fermented products, *L. lactis* is better known for its ability to provide effective means of food preservation. This lactococcal trait derives directly from its simple metabolism, in which sugars are converted into lactic acid and energy is conserved mainly through substrate level phosphorylation. As a consequence of lactic acid production, the pH of the medium drops, leading to a decreased glycolytic flux and growth rate, and ultimately compromising cell viability [7]. Thus, *L. lactis* survival and performance in industrial fermentations are highly dependent on the ability of the organism to respond to acid stress.

Lactic acid has a pK_a_ of 3.86. It is well established that the toxic effect of weak organic acids in bacterial metabolism is primarily dependent on the concentration of the protonated form, which can cross the cell membrane, hence acting as a protonophore [8]. Obviously, the population of the neutral form increases at low pH. For example, at pH 6 the mole fraction of the protonated form of lactic acid is 0.007, whereas at pH 4.8 that fraction increases to 0.103. Therefore, there are two components that should be taken into account when assessing the detrimental effect of low pH on glucose metabolism of *L. lactis*: the effect of the H^+^ concentration, which may lead to disruption of the structures of cell membranes, proteins and other macromolecules, and the negative effect of lactic acid on intracellular pH homeostasis.

The data accumulated during the past two decades indicate that *L. lactis* has developed different strategies to survive and adapt to suboptimal pH conditions [9–11]. Among these mechanisms, the F_0_F_1_-ATPase has been identified as a main player in the maintenance of the pH homeostasis [12]. Additionally, altered expression of proton sinks, such as the citrate-lactate antiporter, and of the arginine catabolic pathway (alkalinisation of the medium by production of NH_3_), have been reported [13,14]. Besides its intrinsic acid resistance, *L. lactis* can reach a physiologic state of increased tolerance to acid when pre-exposed to a moderate acid stress [10,11]. This adaptive response, commonly referred to as the Acid Tolerance Response, was shown to depend on protein synthesis [11]. Indeed, a number of proteome and transcriptome analyses identified several genes involved in the acid regulon of this bacterium, including those related to DNA repair (*dnaK*, *recA*), stringent response (*relA*), and protein folding [9,10,15]. However, there is a clear deficit of metabolic data for the lactococcal acid response. Also, it is frustrating for the scientific community that the identity of the lactic acid exporter(s) in *L. lactis* remains unknown despite the vital role of this system in an organism that converts sugar into lactic acid with nearly 100% yield.

This study represents our efforts to integrate data obtained at the metabolite and transcript levels in response to pH variations. To better understand the system response to the pH perturbation and discriminate between metabolic and transcriptional effects, we probed glucose metabolism at different external pH values (6.5, 5.5, 5.1 and 4.8) in non-growing cells cultivated at the optimal pH of 6.5 (direct effects of acid stress on metabolism), or grown at pH 5.1 and metabolizing glucose also at pH 5.1 (direct effects plus transcriptional adjustment to low pH). Time series data on metabolite pools during the metabolism of glucose were acquired non-invasively by using NMR. The concentrations of glucose, phosphorylated intermediates, and the intracellular and extracellular lactic acid pools were obtained by ^13^C-NMR with a temporal resolution of 30 seconds. Additionally, variations in intracellular pH during the metabolism of glucose were monitored by using ^31^P-NMR. These data permit the calculation of the concentrations of hydrogen ions, lactate and lactic acid in the cytoplasm and in the external medium. The direct effects of pH and of external and internal lactic acid on the glucose transport step were determined by radio-labelled assays in whole cells. The data at the metabolite level were complemented by performing a genome-wide transcriptome analysis, in which transcript levels of cells grown at pH 6.5 were compared to those of cells grown at pH 5.1. The results allowed us to determine the H^+^/lactate stoichiometry of lactic acid export and to propose a metabolic model accounting for the molecular mechanisms underlying the acid stress response in *L. lactis.*

## Materials and Methods

### Bacterial Strains and Growth Conditions

*L. lactis* MG1363 [16] was grown in a 2-liter fermenter in chemically defined medium (CDM) [17] containing 1% (wt/vol) glucose, at 30 ^°^C with pH controlled at 6.5, 5.1 or 4.8. In the fermenter, pH was kept constant by automatic addition of 10 M NaOH. To assure an anaerobic atmosphere, the medium was aseptically gassed with argon during 1 h preceding inoculation (4% inoculum with a culture grown overnight in CDM, initial pH 6.5). Growth was monitored by measuring the optical density at 600 nm (OD_600_). Specific growth rates (μ) were calculated through linear regressions of the plots of ln(OD_600_) versus time during the exponential growth phase. Independent growth experiments were repeated at least three times and the error for each time-point was below 8%.

### *In vivo* NMR Studies with Resting Cells

Cells were grown as described above, harvested during mid-exponential phase and prepared as described elsewhere [18], except that the washing steps were performed with buffer at the pH of the experiment (see below). In brief, for ^13^C-NMR experiments, cells were washed twice with 5 mM potassium phosphate (KP_i_) and suspended in 50 mM KP_i_ buffer (pH 6.5, 5.5, 5.1, or 4.8), to a protein concentration of approximately 15 mg protein ml^-1^. For ^31^P-NMR experiments, cells were washed twice with 10 mM 2-[N-morpholino] ethane-sulfonic acid (MES) buffer and suspended in 50 mM MES buffer (pH 6.5, 5.5, 5.1, or 4.8), to the same protein concentration as for ^13^C-NMR experiments. *In vivo* NMR experiments were performed at pH 6.5, 5.5, 5.1 or 4.8 (pH controlled throughout the experiment by automatic addition of 2 M NaOH, and 30 ^°^C using the circulating system described previously [18,19]. ^13^C- and ^31^P-NMR spectra were acquired at 125.77 MHz and 202.48 MHz, respectively, using a quadruple nuclei probe head at 30 ^°^C on a Bruker AVANCE II 500 MHz spectrometer (Bruker BioSpin GmbH, Karlsruhe, Germany) as described previously [18]. ^13^C-NMR assays were carried out using glucose specifically labelled at carbon one (40 mM) as substrate. After substrate exhaustion and when no changes in the resonances due to end products were observed, a 6 ml-aliquot of cell suspension was passed through a French press (twice at 120 MPa); the resulting extract was incubated at 80 ^°^C (10 min) and cooled down on ice. Macromolecules and cell debris were removed by centrifugation (30,000 *g*) and the supernatant solution, herein designated as “NMR-sample extract”, was used for metabolite quantification [20]. The lactic acid produced was quantified in the NMR-sample extract by ^1^H-NMR on a Bruker AMX300 spectrometer (Bruker BioSpin GmbH). The concentrations of other metabolites were determined in fully relaxed ^13^C spectra of the NMR-sample extracts as previously described [20]. Due to fast pulsing conditions used for acquiring *in vivo* ^13^C-spectra, a direct correlation between concentrations and peak intensities is not feasible. Therefore, correction factors that allow for the determination of intracellular concentrations were used. The correction factors of 0.58 and 0.57 were determined for intracellular and extracellular lactic acid, respectively, after glucose metabolism at pH 4.5. Under these conditions, the intensity of the resonances due to intra- and extracellular lactate remained constant, enabling the acquisition of fully and partially relaxed spectra and the determination of correction factors for saturation and nuclear Overhauser effects. Intracellular pH values were determined from the chemical shift of the resonance due to intracellular phosphate by using a suitable calibration curve (inorganic phosphate chemical shift *versus* pH) obtained with a solution containing 5 mM KP_i_, 50 mM MES buffer, 85 mM NaCl and 5 mM MgCl_2_. Phosphoric acid (85%) contained in a capillary tube was used as a chemical shift reference.

For dry-mass determination, cells were harvested by filtration through 0.22-µm pore-size membranes, and dried to constant mass at 100 ^°^C. Intracellular concentrations of metabolites were calculated using 1.67 µl per mg cell dry weight for the intracellular volume of *L. lactis* [21]. Typically, each NMR experiment was repeated three times.

### Deconvolution of Overlapping Intra- and Extracellular Lactic Acid Resonances

Partial overlap of the resonances due to intracellular and extracellular lactic acid hampered direct integration. Therefore, to estimate the areas of intracellular and extracellular lactic acid resonances, deconvolution was performed by fitting a sum of two hybrid Lorentzian/Gaussian functions to the relevant spectral region, using Matlab V 7.9 (The MathWorks, Inc., Natick, MA, USA).

### Transport Assays with Radio-Labelled Glucose

*L. lactis* was grown at pH 6.5 as described above. Cells were harvested during mid-exponential phase of growth, washed twice with KP_i_ buffer (5 mM, pH 6.5, 5.5, 5.1 and 4.8) and suspended in 50 mM KP_i_ buffer at the same pH as for the washing steps. To investigate the effect of extracellular lactic acid on glucose uptake, cells were suspended in 50 mM KP_i_ buffer containing 60 mM sodium lactate, at the final pH values of 6.5, 5.5, 5.1 and 4.8. To assess the effect of intracellular lactate on glucose uptake, *L. lactis* cells were grown at pH 6.5 and prepared as described for *in vivo* NMR studies (see above). To load cells with lactic acid, glucose (40 mM) was added to a suspension kept at pH 6.5. After 40 min of incubation, samples were diluted 80 times in fresh KP_i_ buffer (50 mM) supplemented with lactate (60 mM) at pH 6.5, 5.5, 5.1 and 4.8, and further incubated for 40 min at 30 ^°^C prior to the transport assays. Initial glucose uptake rates were measured at 30 ^°^C in 100 µl cell suspensions with OD_600_ of 0.5 (to study the high affinity transport) or 2.0 (to study the total transport capacity) [22]. The radio-labelled glucose solution was prepared in KP_i_ buffer (50 mM) with or without lactate (60 mM) at pHs 6.5, 5.5, 5.1 and 4.8. Uptake was stopped by adding 1.5 ml of ice-cold LiCl (0.1 M) immediately followed by filtering through a 0.45 µm nitrocellulose filter (Millipore, Bedford, MA, USA). The high affinity transport was assayed by using a 0.2 mM glucose solution containing [U-^14^C] glucose to give a specific radioactivity of 12.5 µCi µmol^-1^. The final concentration of glucose in the cell suspension was 0.1 mM and transport was allowed to proceed for 5 s. The total transport capacity (low and high affinity transporters) was assayed in the same way except that the final glucose concentration was 10 mM with a specific radioactivity of 0.5 µCi µmol^-1^ and the uptake was stopped at 10 s after glucose addition. This long time was dictated by the low affinity of the transporters, which implied the use of a lower specific radioactivity. Further increase of cell density was impractical.

The filters were washed with 10 ml of KP_i_ buffer (50 mM, pH 6.5), submerged in 5 ml of scintillation liquid cocktail (PerkinElmer, Waltham, MA, USA), and the radioactivity was counted in a LS-6500 scintillation counter (Beckman Coulter, Fullerton, CA, USA). Unspecific binding was determined by adding the same radio-labelled glucose solution to the cell suspension immediately after addition of ice-cold LiCl. The assays were performed in triplicate using cells from at least two independent cultures.

### Transcriptome Analysis

*L. lactis* was grown as described above and cell suspensions were prepared as described for *in vivo* NMR studies. The levels of mRNA of cells grown at pH 6.5 and suspended in 50 mM KP_i_ buffer, pH 5.1 and MG1363 grown at pH 5.1 and suspended in 50 mM KP_i_, pH 5.1 were compared by transcriptome analysis using full-genome amplicon-based *L. lactis* MG1363 DNA-microarrays [23]. Additionally, transcripts of cells grown at pH 6.5 and suspended in 50 mM KP_i_ buffer at pH 6.5 were compared by transcriptomics with the levels of mRNAs in MG1363 grown at pH 5.1 and suspended in 50 mM KP_i_ buffer, pH 5.1. Comparison of the data from the two sets of experiments permits assessment of the changes in gene expression that are influenced by pH, in the absence of cell growth (non-growing suspensions). The experiments were performed essentially as described by van Hijum et al. [24], with the modifications introduced by Pool et al. [25]. The assays were performed in duplicate using cells from two independent cultures. Genes were considered to have significantly altered expression when the Bayesian *p*-value was <0.001, the false discovery rate was <0.01 and the ratio (signal adjusted cells/signal non-adjusted cells) was >2. The microarray data were submitted to the GEO database (GSE47012).

### Analysis of Intracellular Potassium and Sodium Concentrations

*L. lactis* was grown at pH 6.5 as described above and cell suspensions were prepared as described for *in vivo* NMR studies. Cells were allowed to metabolise glucose (40 mM) for 40 min at constant pH of 5.1. Samples (1 ml) were taken at time points zero (immediately before glucose addition), and at 40 min (glucose depleted). Intracellular potassium and sodium concentrations were measured using silicone oil centrifugation [26]. Cell samples were centrifuged (13200 rpm, 15 min) through 0.3 ml of silicone oil, and the pellet was extracted with 1 M perchloric acid (1 ml) overnight at room temperature. After a second centrifugation, the potassium and sodium concentrations were determined by Flame Atomic Absorption Spectrometry using an Analytic Jena AG-AAS5FL (REQUIMTE Laboratory of Analysis, FCT-UNL, Lisboa, Portugal).

## Results

### Effect of pH on the Growth Properties of *L. lactis* MG1363

**Figure 1:**
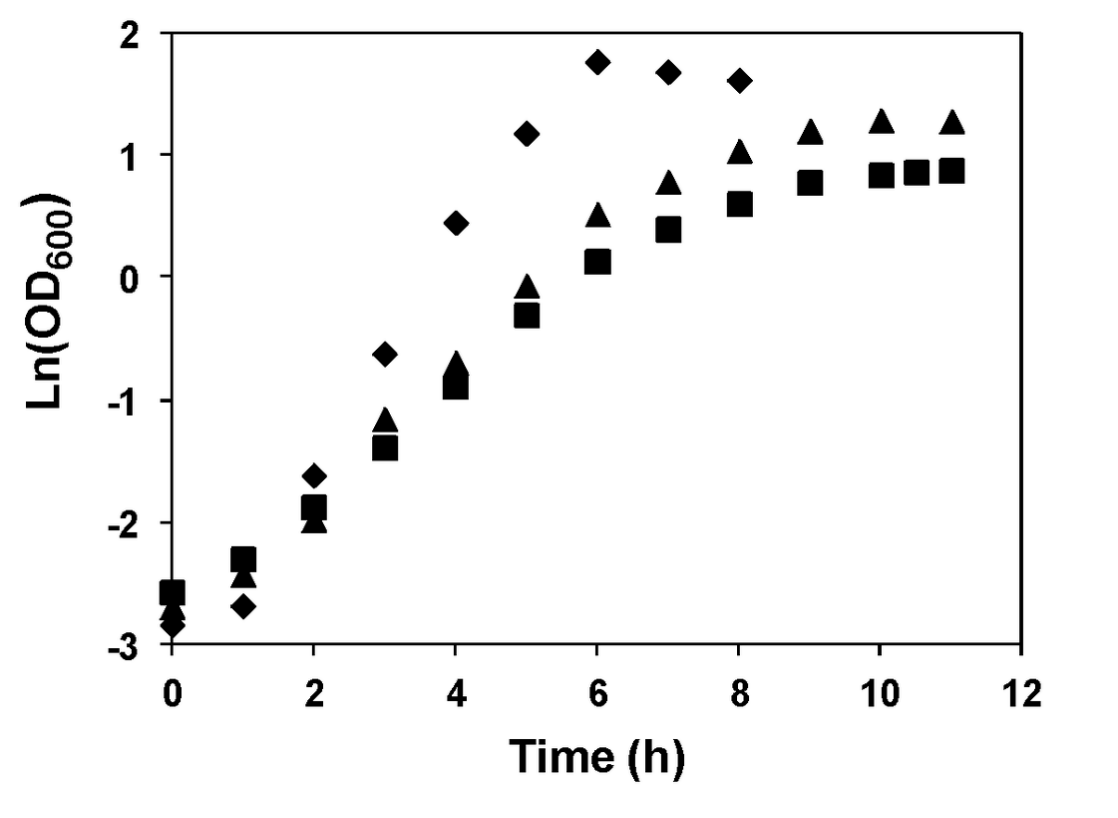
Growth profiles of *L. lactis* at different pH values. Growth was performed in CDM with 1% glucose (w/v) at 30 ^°^C, with pH controlled at 6.5, 5.1 and 4.8. pH was controlled by addition of 10 M NaOH. Growth rate (μ): pH 6.5, 0.97±0.08 h^-1^; pH 5.1, 0.63±0.04 h^-1^; pH 4.8, 0.53±0.01 h^-1^. Symbols: diamond, pH 6.5; triangle, pH 5.1; square, pH 4.8. Data shown are representative from at least two identical experiments.

It is well established that the growth of *L. lactis* is affected by pH [27]. In this work, *L. lactis* MG1363 was grown in the CDM optimized for *in vivo* NMR experiments (devoid of paramagnetic ions), at controlled pHs of 6.5, 5.1 and 4.8 (Figure 1). At the optimal pH of 6.5, the maximal OD_600_ of 5.6±0.3 was reached in approx. 6.5 h, with the cells growing at a specific growth rate of 0.97±0.08 h^-1^. As expected, *L. lactis* cells growing at pH 5.1 presented a lower maximal OD_600_ (3.8±0.3) and specific growth rate (0.63±0.04 h^-1^). This behaviour was even more pronounced at pH 4.8 (maximal OD_600_ of 2.35±0.04 and µ_max_ of 0.53±0.01 h^-1^). Cultures grown at pH 6.5 and 5.1 were used for in-depth metabolic studies.

### Effects of pH on Glucose Metabolism

**Figure 2:**
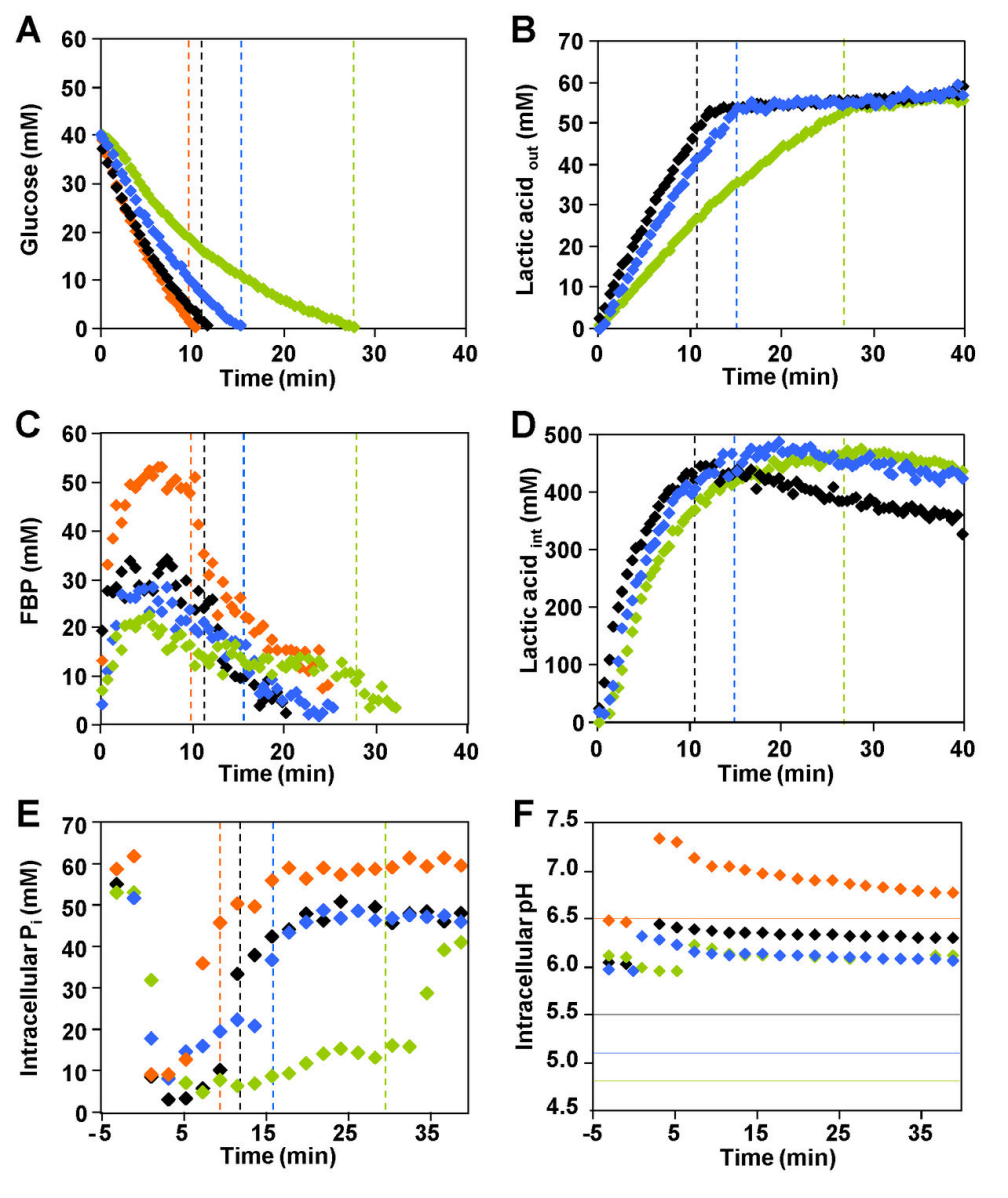
Time courses obtained by *in vivo* NMR during glucose metabolism in *L. lactis*. A, B, C and D: time courses obtained during the metabolism of [1-^13^C] glucose in non-growing cells of *L. lactis* as monitored online by *in vivo* ^13^C-NMR. E and F: biochemical parameters determined during the metabolism of 40 mM glucose in *L. lactis* MG1363 as monitored online by *in vivo* ^31^P-NMR. The experiments were carried out at 30 ^°^C, under anaerobic conditions and pH controlled at 6.5 (orange diamonds), 5.5 (black diamonds), 5.1 (blue diamonds) and 4.8 (green diamonds). (A) Kinetics of [1-^13^C] glucose (40 mM) consumption, (B) extracellular pool of lactic acid, (C) pools of FBP (fructose 1,6-bisphosphate), (D) profiles of intracellular lactic acid, (E) profiles of intracellular P_i_, and (F) intracellular pH. The time points for glucose exhaustion are indicated by vertical dashed lines (graph A, B, C, D and E). The horizontal lines in graph F represent the constant values at which the extracellular pH was controlled. The lack of information on intra and extracellular concentrations of lactic acid at pH 6.5 is due to severe overlap of the lactic acid resonances at this pH and consequent large uncertainty in the measurements of individual areas. Each experiment was performed at least twice with good reproducibility.

The kinetics of [1-^13^C] glucose consumption, product formation and the pools of intracellular metabolites were investigated in cell suspensions derived from cultures grown at optimal pH (6.5) and suspended in working buffer at the selected pH values of 6.5, 5.5, 5.1 and 4.8 (Figures 2A, 2B, 2C and 2D). At pH 6.5 (optimal pH), glucose was consumed at a maximal rate of 0.38±0.03 µmol min^-1^ mg protein^-1^. We observed a direct relationship between pH and maximal substrate consumption rate: glucose was consumed at 0.30±0.04, 0.22±0.02 and 0.16±0.01 µmol min^-1^ mg protein^-1^ when the external pH was 5.5, 5.1 or 4.8, respectively (Figure 2A). For all pH conditions, glucose metabolism was fully homolactic, with lactic acid accounting for approximately 96% of the glucose consumed (Figure 2, panels B and D). Minor amounts of ethanol, 2,3-butanediol, and acetate were also produced (data not shown).

**Figure 3:**
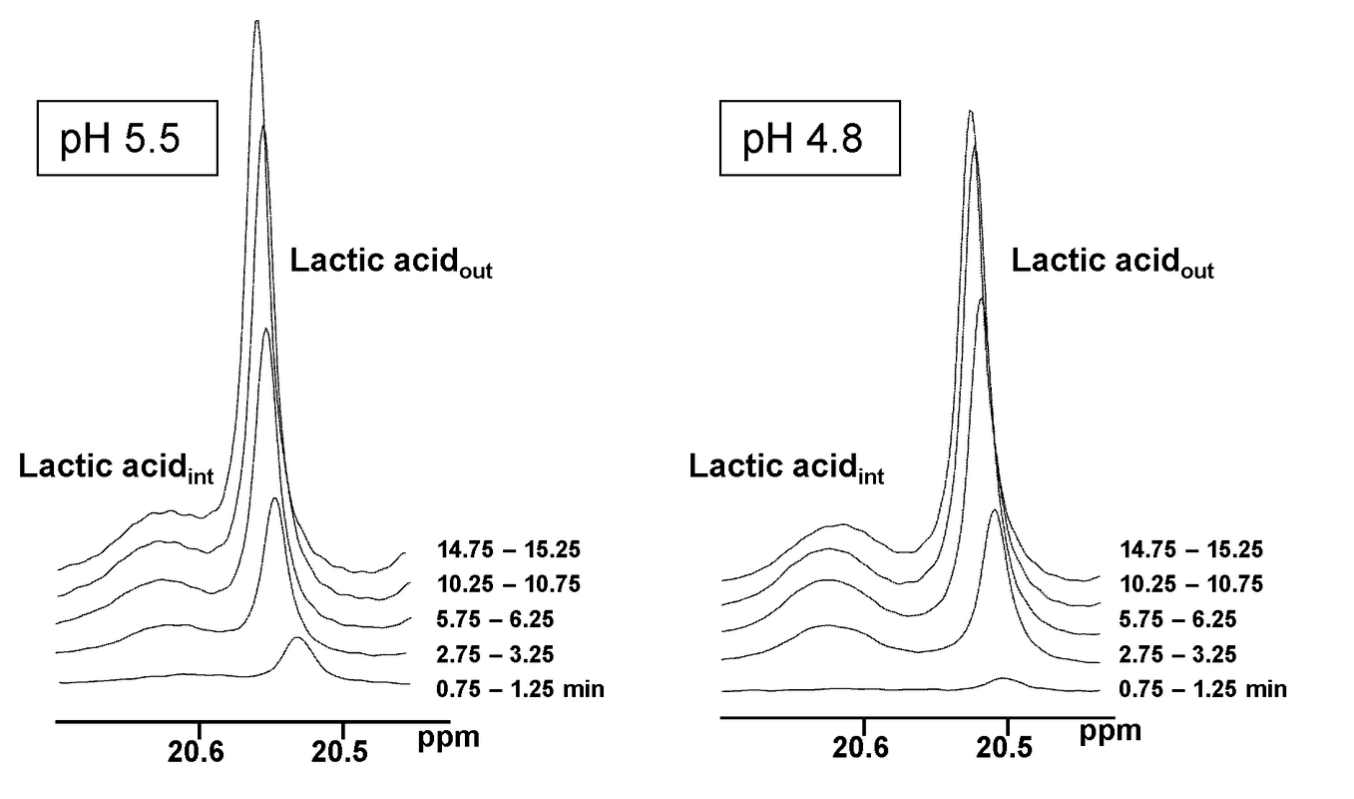
Sequences of ^13^C-NMR spectra of *L. lactis* cell suspensions (pH 4.8 and 5.5), showing separate resonances due to intra- and extracellular lactic acid. Due to the fast rate of proton exchange each resonance represents the total contribution of the two forms of lactic acid (dissociated and non-dissociated forms). The extracellular pH varies by about 0.2 units because of a lag in the addition of base in the circulating system used for the NMR experiment.

In the ^13^C-NMR spectra of *L. lactis* suspensions metabolizing 40 mM [1-^13^C] glucose, two resonances were assigned to the methyl group (carbon 3) of intracellular and extracellular lactic acid (Figure 3). The different pH values of the intracellular and extracellular compartments, and consequently the different degree of lactic acid dissociation, led to distinct NMR resonances arising from the intracellular and extracellular pools of this organic acid. For each pH examined, the profile of extracellular lactic acid followed a trend that mirrors that of glucose depletion (compare panels A and B in Figure 2), with the rate of lactic acid accumulation appreciably decreasing with pH (about 2.4 times from pH 5.5 to 4.8). Interestingly, the accumulation profile of intracellular lactic acid was less sensitive to the buffer pH than its extracellular counterpart, with the accumulation rate decreasing only by a factor of 1.6 when the external pH was lowered from 5.5 to 4.8 (Figure 2D). The intracellular lactic acid pools reached maximal concentrations of about 500 mM at low pH’s (5.5, 5.1 and 4.8). It is important to note that the measurements at external pH 6.5 are not shown as they have a high uncertainty due to the small difference between the pH’s of the internal and external compartments and consequent deficient separation of the respective NMR signals.

At optimal pH 6.5, the dynamics of glycolytic metabolite pools was similar to previous results described for *L. lactis* strains MG1363 and NZ9000 (Figure 2C) [20,22]. Decreasing the pH had a clear effect on the pools of glycolytic metabolites (Figure 2C and Table 1). As for pH 6.5, fructose 1,6-bisphosphate (FBP) accumulated transiently, but the maximal concentration and the profile of depletion depended on the external pH. The pools of 3-phosphoglycerate (3-PGA) and phosphoenolpyruvate (PEP) were also pH dependent, and their maximal concentrations decreased with the pH of the external medium (Table 1). Trehalose 6-phosphate (T6P) was detected transiently only at suboptimal pHs (5.5, 5.1 and 4.8). Minor resonances due to UDP-glucose were observed at all pHs examined, but reliable quantification was hampered by the vicinity of the strong glucose resonance.

**Table 1:** Maximal levels of glycolytic intermediates and glucose consumption rate during glucose metabolism by non-growing cells of *L. lactis* MG1363 at different pH conditions.

**Growth pH**	**External pH**	**FBP_max_ (mM)**	**3-PGA (mM)**		**PEP (mM)**	**T6P (mM)**	**GCR_max_ (µmol min^-1^ mg protein ^-1^)**
6.5	6.5	46.6±3.8	9.1±1.5	6.5±0.4^#^		ND	0.38±0.03
	5.5	33.3±3.0	6.0±1.8	ND		1.6±0.5	0.30±0.04
	5.1	27.6±1.3	5.1±1.2^#^	ND		1.4±0.1	0.22±0.02
	4.8	22.4±1.8	ND	ND		1.0±0.1	0.16±0.01
5.1	5.1	37.5±6.7	5.2±1.0^#^	ND		ND	0.34±0.05

The values are averages of at least two independent experiments.

# Values determined by ^13^C-NMR in crude cell extracts obtained from the cell suspensions used in the *in vivo* NMR experiments. ND, not detected *in vivo* and in cell extracts.

### Information from ^31^P-NMR Analysis

The levels of nucleoside triphosphates (NTP) and intracellular inorganic phosphate (P_i_), as well as the time course of intracellular pH were monitored during the metabolism of glucose (40 mM) by *in vivo* ^31^P-NMR (Figure 2, E and F). For all pH conditions (6.5, 5.5, 5.1, and 4.8), NTP accumulated transiently while glucose was available, reaching maximal concentrations of approximately 8 mM at pHs 6.5 and 5.5, and 5 mM at pHs 5.1 and 4.8. The intracellular pool of inorganic phosphate followed a trend roughly opposite to that of FBP (compare panels E and C in Figure 2; note different *x*-axes), decreasing abruptly when glucose was supplied and recovering to values of the order of 50 mM upon glucose depletion.

Irrespective of the extracellular pH, the intracellular pH increased immediately and in a transient fashion upon glucose addition, but the magnitude of the pH jump decreased progressively with the external pH (Figure 2F). In energized cells the maximum ΔpH, i.e., the difference between intra- and extracellular pH, varied between 0.7 units at optimal pH and 1.3 units at an external pH of 4.8.

### Effect of External pH and Lactic Acid on Glucose Transport

**Figure 4:**
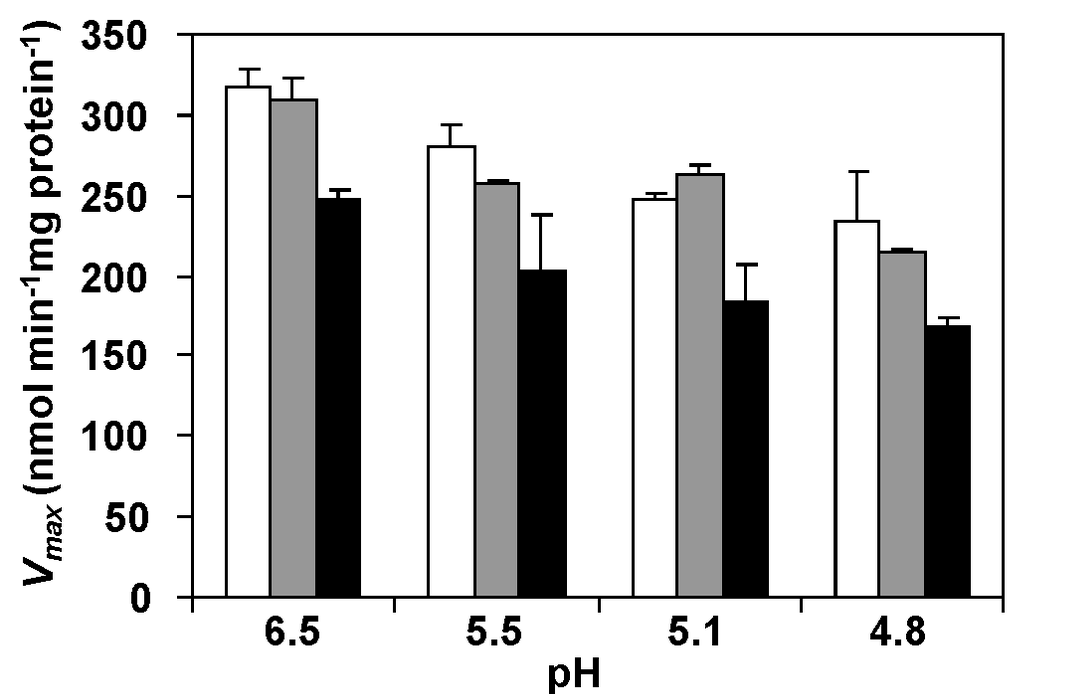
Effect of various conditions on the maximal glucose uptake rate in *L. lactis*. Effect of pH alone (white bars), extracellular lactic acid (grey bars) and intra and extracellular lactic acid (black bars) on *V*_max_ (nmol min^-1^ mg protein^-1^) for glucose uptake assessed by [^14^C] radio-labelled assays with *L. lactis* cells. Glucose was added to a final concentration of 0.1 mM.

To assess the direct effect of pH on the uptake of glucose, transport assays were performed with *L. lactis* MG1363 grown at pH 6.5. Cells were suspended in buffer at the same pHs used for *in vivo* NMR experiments, i.e., 6.5, 5.5, 5.1 or 4.8. *L. lactis* can take up glucose via the high affinity transporter PTS^Man^, and the low affinity transporters PTS^Cel^ and GlcU [25]. Thus, glucose at concentrations of 10 and 0.1 mM were used to discriminate between the effect of pH on the low and high affinity components, respectively. At optimal pH (6.5), the apparent *V*_max_ found for the high affinity transport was 317±13 nmol min^-1^ mg^-1^ protein. *V*_max_ decreased linearly with the pH (Figure 4, white bars); at an external pH of 4.8, the *V*_max_ was 70% of the apparent maximal rate observed at pH 6.5. At each pH the values for the apparent *V*_max_ were similar, regardless of glucose concentration, suggesting that the low affinity transporters do not make a detectable contribution to the uptake of glucose (data not shown). Moreover, transport assays carried out with 0.1 mM glucose in the presence of sodium lactate (60 mM), showed that the apparent *V*_max_ were similar to those determined in the absence of lactate (Figure 4, grey bars). However, when these experiments were performed in cells preloaded with lactate, a reduction of about 25% was observed in the apparent *V*_max_ at each pH (Figure 4, black bars).

### Characterization of Glucose Metabolism in *L. lactis* Pre-Adjusted to Acid Stress

**Figure 5:**
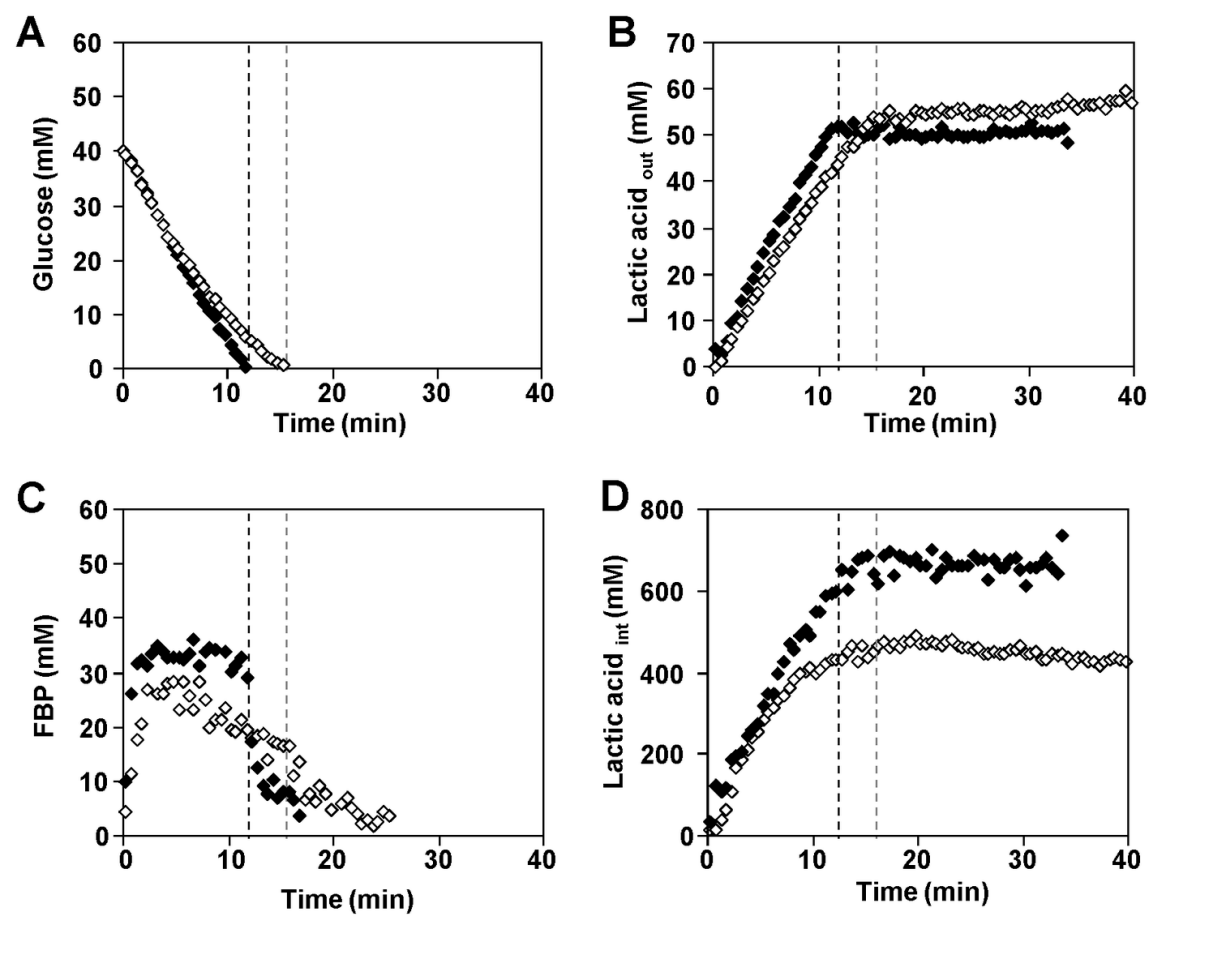
Metabolite time series obtained during glucose metabolism in non-growing cells of *L. lactis*. Cells grown at 6.5 (open diamonds) or 5.1 (filled diamonds). The metabolite pools were monitored online by *in vivo* ^13^C-NMR at 30 ^°^C, under anaerobic conditions and pH controlled at 5.1 in both cases. (A) Kinetics of [1-^13^C] glucose (40 mM) consumption, (B) extracellular lactic acid formation, (C) pools of fructose 1,6-bisphosphate, and (D) profiles of intracellular lactic acid. The glucose consumption rate was 0.22 and 0.34 µmol min^-1^ mg protein^-1^ for non-adapted and adapted cells, respectively. Each experiment was performed twice with good reproducibility.

The kinetics of [1-^13^C] glucose consumption, product formation and the pools of intracellular metabolites of *L. lactis* grown under acid stress conditions (pH 5.1), were monitored *in vivo* by NMR in cell suspensions metabolizing glucose at a constant external pH of 5.1 (Figure 5). These pre-adjusted cells (hereafter designated as adapted cells), consumed glucose at a maximal rate of 0.34±0.05 µmol min^-1^ mg protein^-1^, a value 1.3-fold higher than in non-adapted cells (grown at pH 6.5 and suspended in buffer at pH 5.1) (Figure 5A). Moreover, FBP accumulated transiently to a maximal concentration of 37.5±6.7 mM, about 10 mM greater than the pool size in non-adapted cells (Figure 5C). Lactic acid was the main product of glucose metabolism by adapted cells, as for non-adapted cells, accounting for 96% of the glucose consumed, (Figure 5B). Intracellular lactic acid accumulated immediately after glucose addition reaching a concentration of about 700 mM by the time of glucose exhaustion, a value higher than that determined with non-adapted cells (Figure 5D). This high intracellular concentration remained fairly stable after glucose depletion.

The levels and profiles of nucleoside triphosphates (NTP) and intracellular P_i_ as well as the evolution of intracellular pH of adapted lactococcal cells (grown at pH 5.1) resembled those of non-adapted cells (grown at pH 6.5) in all aspects during the metabolism of glucose at pH 5.1 (data not shown).

To evaluate whether the differences in the metabolic profiles (Figure 5) observed for cell suspensions metabolizing glucose at pH 5.1, grown at pH 5.1 or 6.5, were due to transcriptional variations, a DNA microarray analysis was performed. Using full-genome *L. lactis* DNA microarrays, we compared the mRNA levels of non-adapted (grown at pH 6.5) versus adapted (grown at pH 5.1) MG1363 cells suspended in KPi (50 mM, pH 5.1). Among the 2597 genes in the *L. lactis* MG1363 chromosome, about 8% were differentially expressed when comparing adapted and non-adapted cells. Of these, 132 genes (5%) were up-regulated, while 78 (3%) were repressed in response to acid stress (Tables S1 and S2).

Expression of genes related to carbohydrate metabolism, energy conservation and stress response was analyzed in detail (Table 2). Interestingly, various genes directly involved in glucose catabolism were overexpressed between 2- and 3-fold in cells grown at pH 5.1. The overexpression of *ptcC*, which encodes the glucose transport protein PTS^cel^, and of some other genes encoding glycolytic enzymes such as, *gapA* and *pyk* (Table 2), fit well with the higher glucose consumption rate observed for adapted cells. Also, various stress response related genes were differently expressed in cells grown at low and optimal pH (pH 5.1 and 6.5, respectively), such as those encoding the chaperones DnaK and GroEL and the stringent stress signal RelA. The overexpression of all subunits of H^+^-ATPase (2.2-3.0 times) in adapted cells agrees with previous studies on the acid stress response of *L. lactis* [9,12].

**Table 2:** Differentially expressed genes in *L. lactis* MG1363 cell suspensions (pH 5.1) derived from cultures grown at pH 5.1 (acid stress) or 6.5 (optimal pH).

**Function**	**Gene**	**Fold change^*a*^**	**Description of gene product**
Carbohydrate metabolism and energy conservation	*atpC*	2.8	F_0_F_1_ ATP synthase subunit epsilon
	*atpD*	3.0	F_0_F_1_ ATP synthase subunit beta
	*atpG*	2.9	F_0_F_1_ ATP synthase subunit gamma
	*atpA*	2.2	F_0_F_1_ ATP synthase subunit alpha
	*atpF*	2.5	F_0_F_1_ ATP synthase subunit B
	*atpB*	2.9	F_0_F_1_ ATP synthase subunit A
	*atpE*	2.2	F_0_F_1_ ATP synthase subunit C
	*ldh*	2.6	L-lactate dehydrogenase
	*ptcC*	2.4	Cellobiose-specific PTS system IIC component
	*gapA*	2.4	Glyceraldehyde 3-phosphate dehydrogenase
	*eno*	2.6	Enolase
	*gpmA*	2.0	Phosphoglycerate mutase
	*pgi*	2.4	Glucose-6-phosphate isomerise
	*pyk*	2.4	Pyruvate kinase
	*pgk*	2.3	Phosphoglycerate kinase
	*llmg_2513*	6.1	Putative transport protein
Stress response	*recA*	2.0	Recombinase A
	*dnaK*	2.7	Molecular chaperone DnaK
	*clpE*	2.5	ATP-dependent Clp protease ATP-binding subunit ClpE
	*groEL*	2.5	Chaperonin GroEL
	*clpB*	2.1	ATP-dependent Clp protease
	*soda*	2.2	SodA protein
	*relA*	-2.3	GTP pyrophosphokinase
	*llrE*	-2.0	Two-component system regulator llrE

^a^ Positive values indicate up-regulation and negative values indicate down-regulation. Genes with significantly altered expression (p<0.001) and expression ratio higher than |±2.0| are shown.

## Discussion

In this work, the acid stress response of *L. lactis* was investigated at the metabolic and transcriptional levels, with the general aim of providing pointers for the development of strains with improved acid resistance. A more specific goal was to obtain information on features of the yet unknown lactic acid export system. We took advantage of the non-invasive and analytical power of NMR to obtain online metabolic information during glucose utilization in *L. lactis*. To assess the direct effects of the extracellular pH on dynamics of metabolite pools, cell energy status, and glycolytic performance, one group of cells was grown at optimal pH (6.5), then suspended in buffer of different pH values and allowed to metabolize a pulse of glucose. By this means, the effect of acid stress on the performance of the existing cell machinery was studied without the complications arising from alterations in protein levels. A second group of cells was grown under acidic conditions (pH 5.1), and hence allowed to adjust their transcript and protein levels to these stressful conditions. These “adapted cells” were then examined with respect to glucose metabolism under non-growing conditions as for the first group. The non-invasive NMR methodology was especially valuable for providing time series on the intracellular and extracellular pools of lactic acid. To our knowledge, NMR is the only technique able to provide this type of data directly in living cells, without the need of separation/washing steps, which inevitably result in leakage of organic acids and other metabolites.

All experiments designed to study the pH dependence of glucose metabolism were performed with cells grown at the same pH of 6.5; therefore, their transcriptional status is expected to be identical. This was confirmed experimentally: the transcriptional profiles of cells grown at pH 6.5 and suspended in buffer at pH 5.1 were identical to those of cells grown at 6.5 and suspended at pH 6.5 (data not shown). Thus, alterations at the level of gene expression can be ruled out, and the results reflect only the immediate impact of low pH on glucose metabolism, namely on the activity of uptake and export systems and glycolytic enzymes.

The pH of the external medium had a clear effect on the glucose consumption rate (GCR) (Table 1). As the external pH is lowered, the barrier to transporting protons out of the cell increases. Moreover, at lower external pH the proportion of non-dissociated lactic acid is higher and this form may leak into the cell, demanding more energy to preserve pH homeostasis. Additional contributions for impaired glucose consumption at low pH arise from the activity profiles of *L. lactis* glycolytic enzymes, which evolved to perform optimally at pH values near neutrality [28–31], and also from the observed inhibitory effect of low pH on the glucose transport systems (Figure 4). The sensitivity of PTS systems to low pH has been reported for *Streptococcus mutans* [32], hence we studied the pH dependence of glucose uptake in *L. lactis*. Also, we wanted to determine whether the large pools of intracellular lactic acid would affect glucose uptake. We found that the glucose transport is directly inhibited by external [H^+^], as well as by intracellular lactic acid. Therefore, accumulation in the cytoplasm of lactic acid, the end-product of glucose metabolism, exerts negative feedback on glucose import and contributes to the observed slow-down of glucose metabolism at each pH value.

Our transport assays with cells grown at optimal pH also suggest that glucose transport in *L. lactis* MG1363 proceeds primarily through the high affinity system (PTS^Man^), providing no evidence for involvement of the low-affinity transporter as previously proposed for *L. lactis* NZ9000 [22]. This discrepancy could be due to repression of the genes encoding PTS^Cel^ in strain MG1363. In fact, a global transcriptional analysis of strains MG1363 and NZ9000 showed alterations in several transcript levels and, specifically, the expression of gene *ptcC*, encoding the membrane component in PTS^Cel^, decreased 50-fold in MG1363 [33]. The huge accumulation of lactic acid inside the cells as detected by NMR is the most striking feature of our data (Figures 2D and 5D). Within the group of cells grown at optimal pH, the build-up rate of the intracellular lactic acid pool varied between 0.14 and 0.22 µmol min^-1^ mg protein^-1^, approaching a limit at around 500 mM intracellular concentration when glucose was metabolized at sub-optimal pHs (4.8 to 5.5). Surprisingly, the total intracellular pool of lactic acid reached an even higher concentration (approx. 700 mM) in cells that had been grown under acid stress, and the rate of increase remained high until the glucose was exhausted. As the calculation of intracellular concentrations takes the cell volume into account, it was important to verify whether the volume was affected by different experimental conditions. Confocal microscopy was used to confirm that the cell volume was similar in *L. lactis* cells grown at 6.5 and 5.1 and treated as for the NMR experiments. Moreover, no alterations in the cell volume were detected before and after glucose consumption (data not shown). It is also important to consider the concentration of unlabelled lactic acid that could arise from metabolism of carbon reserves in the cells and/or pre-existing intracellular lactic acid. At the end of glucose metabolism, the concentrations of labelled and unlabelled lactic acid were determined by proton NMR. The total amount of lactic acid produced from sources other than the labelled glucose amounted to intracellular maximal concentrations of approx. 120 mM and 150 mM for cells grown at optimal pH and adapted to low pH, respectively. This variation in concentration cannot account for the difference in lactic acid accumulation between non-adapted and acid-adapted cells. Hence, the observed differences in the intracellular lactic acid are significant.

### Stoichiometry of Lactate: Proton Export (*n*)

The measurements of pH_int_ as a function of the imposed external pH (Figure 2F) confirmed the reported high pH homeostasis capacity of *L. lactis* at acidic pH [27,29]; the ΔpH increased strongly with the acidification of the external medium, reaching 1.3 pH units at the external pH of 4.8. At all pH values tested, the intracellular pH was maintained at 6.0 or above. Therefore lactate, the ionized form of lactic acid, should be, by far, the predominant species in the cytoplasm. The internal concentration of lactate was always much higher than in the external medium, which creates a favourable electrochemical gradient for the efflux of lactate. Interestingly, once glucose was exhausted, the intracellular lactate was fairly stable, meaning that the export of lactate is negligible after glucose depletion, despite the favourable concentration gradient. This observation leads to the conclusion that lactate must be exported concomitantly with protons. In the case of a 1: 1 H^+^/lactate stoichiometry, the thermodynamic barrier for lactic acid export is unfavourable under most conditions examined (Figure S1), and it would be still more unfavourable if more than one proton is excreted with lactate, suggesting the need to invoke a mechanism coupled to ATP hydrolysis. This would make it even more important to recover some of the expended energy by allowing proton influx through the ATPase [34].

The observed high intracellular concentration of lactate anion implies an adjustment of the intracellular cation concentration. As KP_i_ buffer was used in the preparation of the cell suspensions and NaOH was used to control pH, potassium and/or sodium were the expected counterions for lactate in the intracellular compartment. However, the increase in intracellular potassium and sodium (50 and 100 mM, respectively, Table S3) does not match the accumulation of around 500 mM lactate ion observed during glucose metabolism. Significantly, the concentrations found for Na^+^ and K^+^ agree well with reported measurements in the same organism [35]. At this stage we are unable to explain how electroneutrality is maintained in the cell.

**Figure 6:**
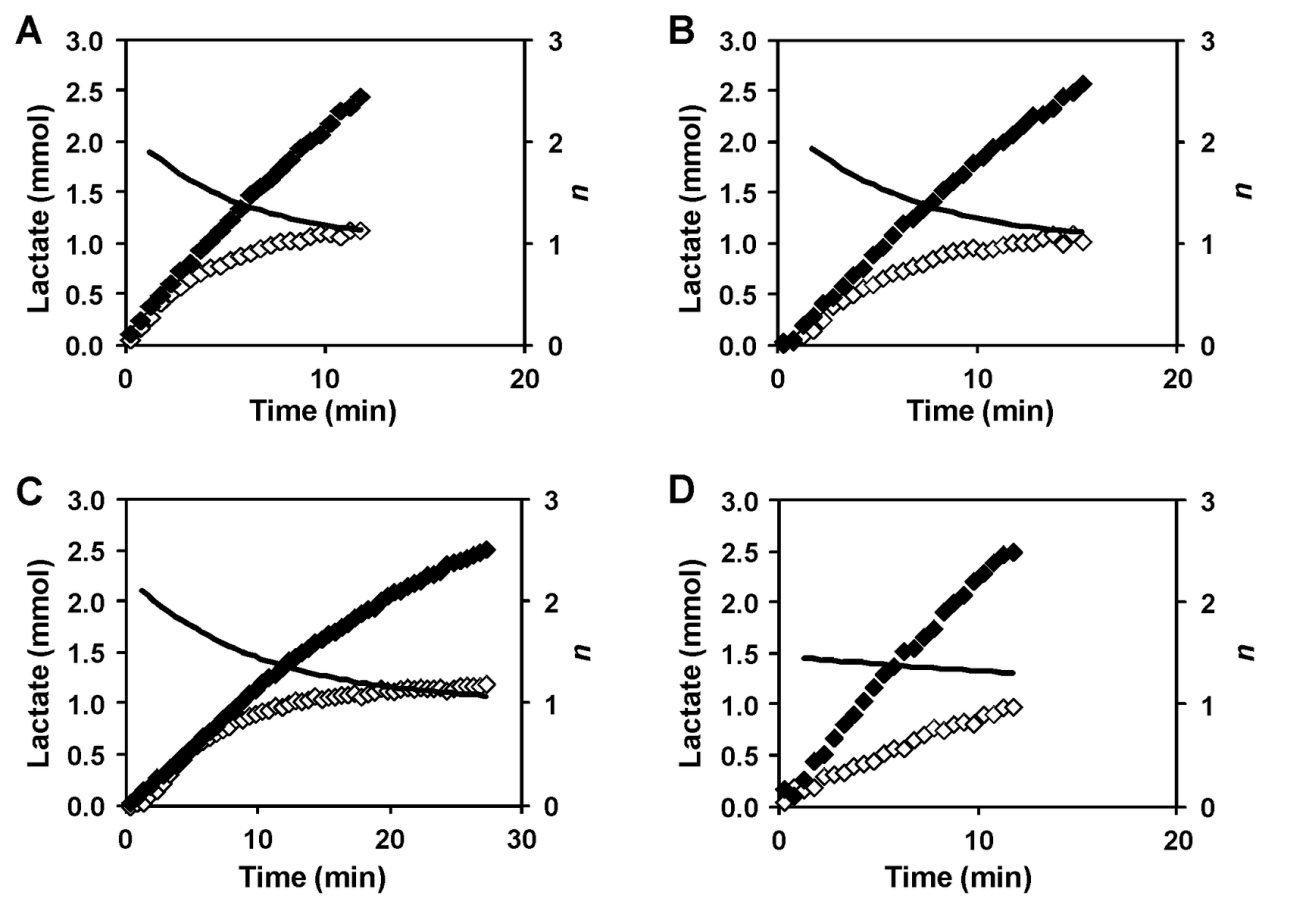
Determination of *n*, the number of protons extruded from the cell concomitantly with each lactate. Profiles of total amounts of internal (open diamonds) and external (filled diamonds) lactate (mmol) in *L. lactis* cell suspensions metabolizing glucose at pH 5.5 (A), 5.1 (B) and 4.8 (C) by cells grown at pH 6.5 or cells at pH 5.1 and previously grown at pH 5.1 (D). The experimental points represented were obtained while glucose was available. The black lines indicate the value of *n*, that was obtained numerically from exponential fits to the intra- and extracellular lactic acid curves.

The high pool of internal lactate suggests that the yet unknown lactic acid exporter(s) must transport lactate in symport with protons. Previous studies calculated *n* (the number of protons exported concomitantly with each lactate), by assuming that the overall driving force for lactic acid transport is close to zero, i.e., in the limit of an equilibrium situation [34,36,37]. In particular, a 1:1 stoichiometry was proposed for lactic acid extrusion in *Lactobacillus helveticus* [38] and in *L. lactis* subsp. *cremoris* at acidic pH [34]. Our data allow the direct determination of *n* since intracellular pH homeostasis implies that one proton is exported for each lactate anion that remains in the cytoplasm. The rate of the build-up of intracellular lactate therefore gives the number of additional protons exported per unit time. Hence, the ratio of the gradients of the build-up of the total amounts of intracellular lactate and extracellular lactic acid (non-dissociated plus dissociated forms) with respect to time is equal to *n*-1 (see Figure 6). The number obtained is *n*-1 simply because the proton of the non-dissociated lactic acid molecule that is exported must also be counted. The values in Figure 6 were obtained by fitting smooth curves to the experimental total pools of lactic acid to obtain the gradients numerically.

In cells grown at pH 6.5 (Figure 6, panels A, B and C), *n* varies between 2 and 1 during glucose consumption, which is not an effect of pH but appears to depend on the lactate concentration. As both intracellular and extracellular lactate increase, the separation of effects is not straightforward. We note that the inhibition of proton pumping from the cell by intracellular lactate would avoid the cell accumulating excessive amounts of this anion that, as discussed above, inhibits glucose uptake. Furthermore, the build-up of intracellular lactate is well fitted by an exponential curve rising to an asymptotic value (see Figure S2), which would occur if the export of additional protons decreased linearly with the intracellular lactate concentration. However, in *L. lactis* subsp. *cremoris* it was concluded that *n*, and hence the amount of intracellular lactate accumulated, depended on the extracellular lactic acid concentration [34]. Therefore, we repeated the NMR experiment at pH 5.1 in the presence of 40 mM external lactic acid (Figure S3). In the experiments without externally added lactic acid, the accumulation of internal lactate had almost ceased when the external lactic acid derived from glucose metabolism reached concentration values ^≈^ 40 mM (see Figure 2, panels B and D). If the internal lactate accumulation were arrested due to external lactic acid then there would be little or no build up of intracellular lactate in the new experiments. In fact, the concentration of intracellular lactate reached levels similar to those observed in the absence of added external lactic acid (compare Figure S3 and Figure 2D). Therefore, the internal build-up of lactate, and concomitant proton extrusion, is definitely unrelated with external lactic acid.

We cannot exclude a possible contribution of the ATPase to proton extrusion, however, since the initial rates of intracellular lactate accumulation are similar for both adapted and non-adapted cells at pH 5.1 (Figure 5D), it is reasonable to assume that the contribution of this pump is similar in both conditions. This is intriguing because the microarray analysis of these cells revealed the overexpression of the ATPase subunits (Table 2). Also, it has been reported that the ATPase activity is 2.8-fold increased when cells are grown at pH 4.5 comparing with pH 7 [12]. As the observed rate of proton extrusion does not correlate with this increase in ATPase activity we propose that the contribution of the ATPase to proton extrusion is not significant and protons are primarily pumped out via the lactic acid exporter(s).

In the adapted cells, *n* appears to be less than 2 at the beginning of glucose consumption but was still greater than 1 when glucose was exhausted (Figure 6D). The reduction in the initial value of *n* might be caused by proton influx through the ATPase in accord with the proposal for energy recycling [34].

The time courses for the total pools of internal and external lactic acid reveal that the intracellular pool starts to level off in non-adapted cells long before glucose was exhausted, whereas it increased almost linearly up to glucose depletion in adapted cells (Figure S4). The slowing down of internal lactate accumulation cannot be due to inhibition of glucose uptake and/or metabolism since the production of external lactic acid is not affected. Rather, it reflects the inhibition of proton extrusion discussed above. The difference in behaviour of adapted cells could be explained by the overexpression of an additional lactic acid exporter that would be less inhibited by the intracellular lactate. The putative additional transporter should have an *n* value that is not strongly dependent on the intracellular lactate concentration (Figure 6D). The transcriptional analysis highlighted a good candidate (llmg_2513), to encode a lactic acid transporter (Table 2). Indeed, this gene encodes a protein that belongs to the major facilitator super-family and has a topology (406 amino acids and 12 transmembranar structures) similar to known lactic acid exporters, such as LldP from *E. coli*, YqkI from *B. subtilis* and JEN1 from *S. cerevisiae* [39–41]. This promising hint led us to construct a *L. lactis* strain in which gene llmg_2513 was deleted. However, this strain showed no significant differences in the profiles of intra and extracellular lactate or in the *n* value (data not shown); therefore, the proposed role of llmg_2513 in lactate export was not confirmed.

### Metabolic Model

The accumulation of trehalose 6-phosphate (T6P) observed in non-adapted cells when metabolizing glucose at sub-optimal pHs indicates a glycolytic constraint, probably at the level of phosphofructokinase (PFK), which would lead to accumulation of glucose 6-phosphate, a precursor for T6P synthesis via β-phosphoglucomutase and T6P phosphorylase, two trehalose catabolizing enzymes present in *L. lactis* [42]. The redirection of carbon flux at the level of glucose 6-phosphate indicates that the negative effect of a decreased internal pH on the glycolytic enzymes must prevail over the impairment of glucose uptake rate at low pH_ext_ (see above). Interestingly, accumulation of T6P was not detected in cells grown at pH 5.1, while non-adapted cells metabolizing glucose at the same pH accumulated 1.4 mM T6P. The distinct behaviours of adapted and non-adapted cells, which are unrelated with the experimental pH as the two groups of cells display identical intracellular (and extracellular) pHs, are reconciled by the observation that both glycolytic enzymes and glucose transporters are upregulated during cell growth at low pH (Table 2).

Adapted cells showed improved metabolic performance at sub-optimal pH. The profiles of glucose depletion, FBP build-up and exhaustion and the lack of T6P accumulation resembled the features of non-adapted cells metabolizing glucose at optimal pH. In other words, during growth at pH 5.1 cells were adjusted to cope optimally with acid stress. Curiously, the glucose uptake in adapted cells was not limited by intracellular lactate, which was clearly observed in non-adapted cells, although the lactate concentration reached the much higher value of 0.7 M, with an extrapolated limit of 1.0 M (Figure S2). The interpretation of these observations relies on transcriptomic data that revealed significant induction of genes encoding glucose transporters (PTS^Cel^), and glycolytic enzymes. It is conceivable that the enhanced H^+^-ATPase activity is an asset to bypass the deleterious effects on pH homeostasis, especially towards the end of fermentation, when the increasing concentration of external lactic acid forces the back-flux of the protonated form into the cytosol to some extent.

**Figure 7:**
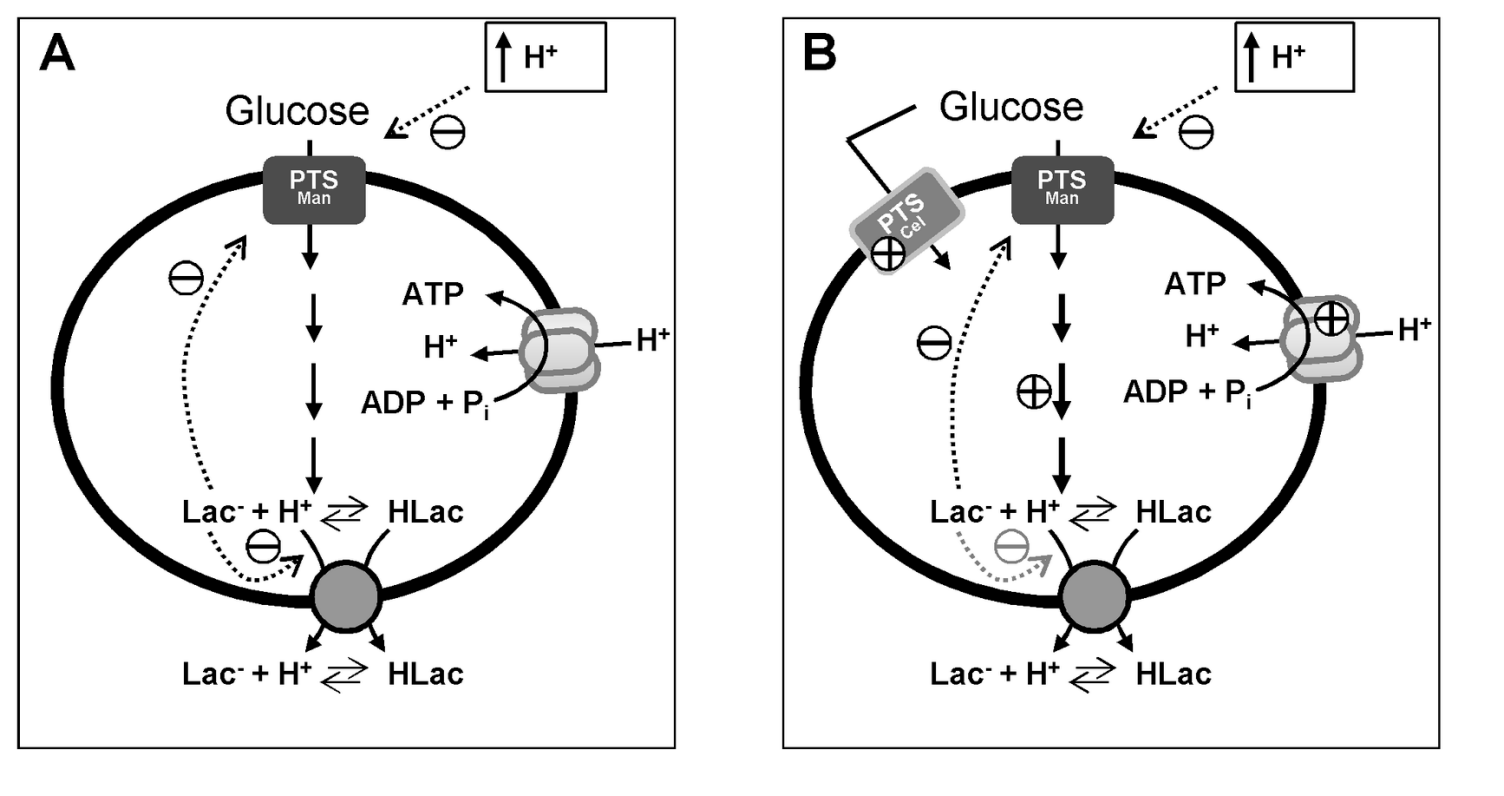
A qualitative model for pH effects and acid stress adaptation of *L. lactis* cells. (A) Non-adapted cells (grown at pH 6.5 and suspended in buffer at pH 5.1); and (B) adapted (grown at pH 5.1 and suspended in buffer at pH 5.1). Dotted arrows indicate metabolic effects with the sign indicated; signs without arrows indicate transcriptional effects. Abbrev: HLac, non-dissociated form of lactic acid; Lac-, lactate anion. The adapted and non-adapted energized cells both had the same internal pH of 6.3.

The metabolic model shown in Figure 7 integrates the set of results obtained in this work. The glucose consumption rate of cells grown at optimal pH decreases with the external pH due to the inhibitory effect of low pH on the activity of the glucose uptake system, combined with the negative effect of lowered internal pH on the activity of glycolytic enzymes. Regardless of the external pH, in the pH range examined (4.8-5.5), the intracellular pool of lactate builds up to maximal values around 0.5 M and lactate accumulation does not depend on the presence of external lactic acid. Furthermore, the pool of internal lactate relative to glucose consumed is fairly independent of pH (Figure S5). The number of protons co-exported with one lactate is also independent of external pH, varying between 2 and 1 during glucose utilization. The process of adaptation to acid stress enables cells to counteract the inhibitory effects mentioned above, through the overexpression of genes encoding glucose transporters, glycolytic enzymes, and additional lactic acid exporter(s) which are less affected by internal lactate. The upregulation of H^+^-ATPase subunits might represent an advantage to growing cells at very acidic pHs. It may also be important for pumping out extra protons that leaked in, or providing an additional capacity to recover energy via proton influx.

## Supporting Information

Figure S1 Ratio of concentrations of lactate anion (open squares) and the inverse ratio of hydrogen ion (filled diamonds). Data obtained for the cytoplasm and the extracellular medium during glucose metabolism at the pH values indicated. The asterisk is used to indicate the data for the cells grown at pH 5.1 (adapted cells). Proton concentrations were calculated according to pH = -log [H^+^]. The extracellular pH was maintained constant and intracellular pH values were determined by ^31^P-NMR *in vivo* (Figure 2F). Lactate anion concentrations were calculated according to pH = pK_a_ + log([Lac^-^]/[HLac]). Extra and intracellular lactate concentrations were monitored online by *in vivo* ^13^C-NMR (Figure 2 B and D). A lactate ratio higher than the hydrogen ion inverse ratio implies that the extrusion of lactic acid from the cell is energetically favourable, which is typically not the case. Vertical dashed lines indicate the time points for glucose exhaustion.
(TIF)

Figure S2 The concentrations of intracellular lactate obtained during glucose metabolism at different pH with solid lines showing fitted exponential curves. The curves fitted for the data obtained at pH 5.5 (black), 5.1 (blue) and 4.8 (green) have a similar asymptotic value (~ 450 mM); whereas the curve that fits the values obtained at pH 5.1 (adapted cells) has an asymptote that is considerably higher (~ 970 mM).
(TIF)

Figure S3 Time courses obtained during the metabolism of 40 mM [1-^13^C] glucose, in the presence of 40 mM [3-^13^C] lactate 50% labelled. The experiment was monitored online by *in vivo* ^13^C-NMR and the pH was controlled at 5.1.Glucose (blue), intracellular lactic acid accumulation (green), extracellular lactic acid production (orange), and transient accumulation of FBP (fructose 1,6-bisphosphate) (red) under anaerobic conditions at pH 5.1. Maximal glucose consumption rate is 0.14 µmol min^-1^ mg prot^-1^.
(TIF)

Figure S4 Profiles of the total amounts of internal (open diamonds) and external (filled diamonds) lactic acid. Data obtained in *L. lactis* cell suspensions metabolizing glucose at the pH values indicated. The asterisk is used to indicate the data for the cells grown at pH 5.1 (adapted cells). Vertical dashed lines indicate the time points for glucose exhaustion.
(TIF)

Figure S5 Profiles of intracellular lactate accumulation as a function of glucose consumed at different pH values. Data obtained during glucose consumption at pH 5.5 (black), 5.1 (blue) and 4.8 (green) as presented in Figure 2 (panels A and D).
(TIF)

Table S1 Genes with significantly higher expression profiles in *L. lactis* strain MG1363 suspended in KPi at pH 5.1, previously adapted to acid (grown at pH 5.1) compared with non-adapted cells (grown at 6.5).
(DOC)

Table S2 Genes with significantly lower expression profiles in *L. lactis* strain MG1363 suspended in KPi at pH 5.1, previously adapted to acid (grown at pH 5.1) compared with non-adapted cells (grown at 6.5).
(DOC)

Table S3 Potassium and sodium content in perchloric extracts of *L. lactis* cells suspensions collected before and after glucose metabolism (40 mM). Cells were grown at pH 6.5 and suspended at pH 5.1. Values represented are averages of two experiments.
(DOC)
