# Supplementary figures and images for: Metabolic and Transcriptional Analysis of Acid Stress in Lactococcus lactis, with a Focus on the Kinetics of Lactic Acid Pools
Source: PLoS One. 2013 Jul 3;8(7):e68470. doi: 10.1371/journal.pone.0068470 (PMC3700934; doi:10.1371/journal.pone.0068470)

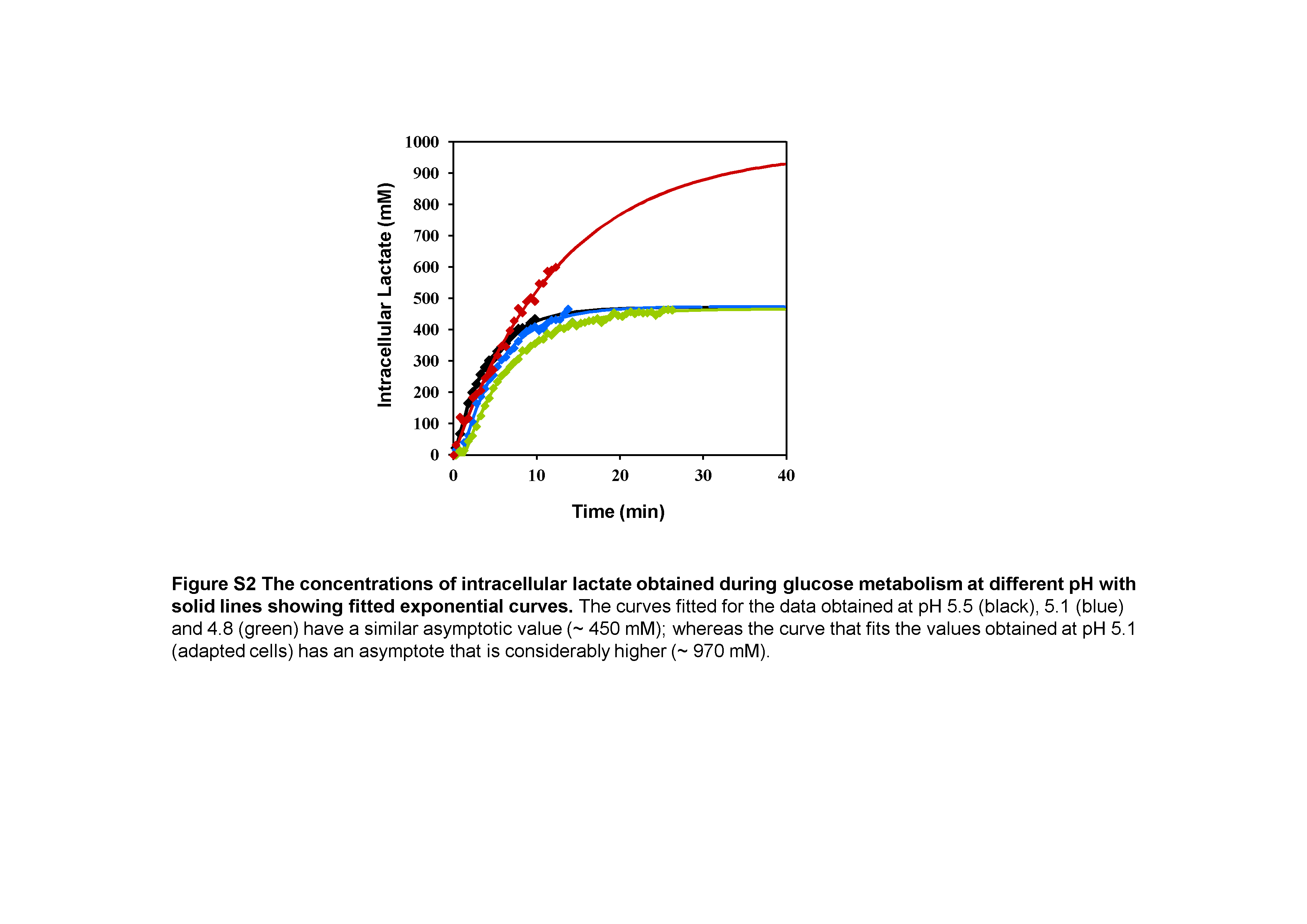

Supplement: Figure S2 — The concentrations of intracellular lactate obtained during glucose metabolism at different pH with solid lines showing fitted exponential curves. The curves fitted for the data obtained at pH 5.5 (black), 5.1 (blue) and 4.8 (green) have a similar asymptotic value (~ 450 mM); whereas the curve that fits the values obtained at pH 5.1 (adapted cells) has an asymptote that is considerably higher (~ 970 mM). (TIF) [file pone.0068470.s002.tif]

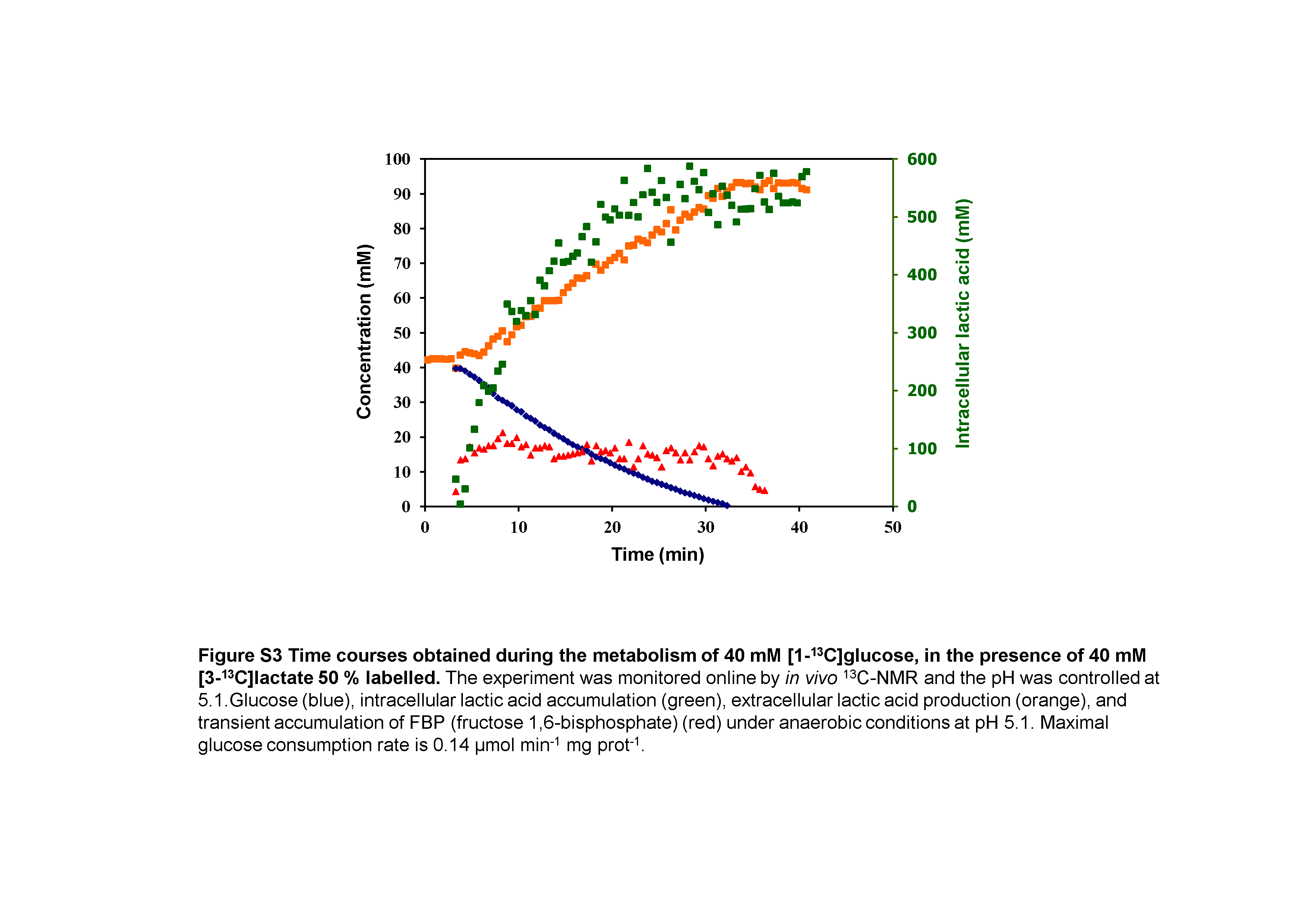

Supplement: Figure S3 — Time courses obtained during the metabolism of 40 mM [1-13C] glucose, in the presence of 40 mM [3-13C] lactate 50% labelled. The experiment was monitored online by in vivo 13C-NMR and the pH was controlled at 5.1.Glucose (blue), intracellular lactic acid accumulation (green), extracellular lactic acid production (orange), and transient accumulation of FBP (fructose 1,6-bisphosphate) (red) under anaerobic conditions at pH 5.1. Maximal glucose consumption rate is 0.14 µmol min-1 mg prot-1. (TIF) [file pone.0068470.s003.tif]

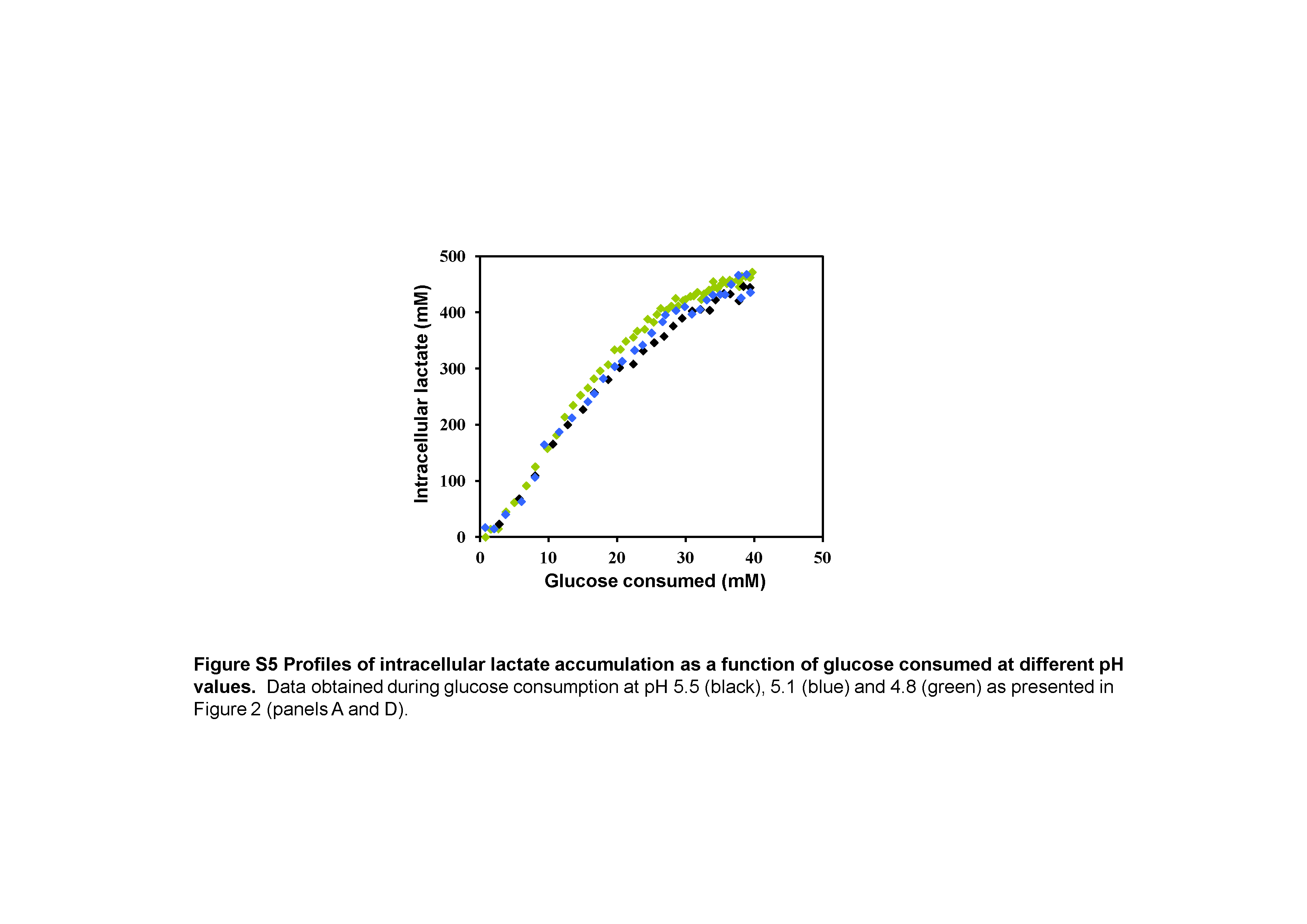

Supplement: Figure S5 — Profiles of intracellular lactate accumulation as a function of glucose consumed at different pH values. Data obtained during glucose consumption at pH 5.5 (black), 5.1 (blue) and 4.8 (green) as presented in Figure 2 (panels A and D). (TIF) [file pone.0068470.s005.tif]
