# Supplementary material for: Metabolic and Transcriptional Analysis of Acid Stress in Lactococcus lactis, with a Focus on the Kinetics of Lactic Acid Pools
Source: PLoS One. 2013 Jul 3;8(7):e68470. doi: 10.1371/journal.pone.0068470 (PMC3700934; doi:10.1371/journal.pone.0068470)
Supplement: Table S1 — Genes with significantly higher expression profiles in L. lactis strain MG1363 suspended in KPi at pH 5.1, previously adapted to acid (grown at pH 5.1) compared with non-adapted cells (grown at 6.5). (DOC) [file pone.0068470.s006.doc]

**Table S1** Genes with significantly higher expression profiles in *L. lactis* strain MG1363 suspended in KPi at pH 5.1, previously adapted to acid (grown at pH 5.1) compared with non-adapted cells (grown at 6.5).

| **Function** | **Gene** | **Fold change** | **Description of gene product** |
| --- | --- | --- | --- |
| Energy production and conversion | gltA | 3.7 | methylcitrate synthase |
|  | icd | 4.0 | isocitrate dehydrogenase |
|  | cydB | 2.0 | cytochrome d ubiquinol oxidase, subunit II |
|  | atpC | 2.8 | F0F1 ATP synthase subunit epsilon |
|  | atpD | 3.0 | F0F1 ATP synthase subunit beta |
|  | atpG | 2.9 | F0F1 ATP synthase subunit gamma |
|  | atpA | 2.2 | F0F1 ATP synthase subunit alpha |
|  | atpF | 2.5 | F0F1 ATP synthase subunit B |
|  | atpB | 2.9 | F0F1 ATP synthase subunit A |
|  | atpE | 2.2 | F0F1 ATP synthase subunit C |
|  | ldh | 2.6 | L-lactate dehydrogenase |
|  | qor * | 2.4 | quinone oxidoreductase |
| Amino acid transport and metabolism | oppA | 2.6 | oligopeptide-binding protein oppA precursor |
|  | - | 5.5 | cysteine synthase |
|  | ilvH | 2.3 | acetolactate synthase 3 regulatory subunit |
|  | hisG | 5.1 | ATP phosphoribosyltransferase catalytic subunit |
|  | hisZ | 4.7 | ATP phosphoribosyltransferase regulatory subunit |
|  | busAB | 2.4 | glycine betaine-binding periplasmic protein precursor |
|  | glnB | 2.7 | nitrogen regulatory protein P-II |
|  | hisI | 3.2 | bifunctional phosphoribosyl-AMP cyclohydrolase/phosphoribosyl-ATP pyrophosphatase protein |
|  | hisF | 3.0 | imidazoleglycerol-phosphate synthase cyclase |
|  | hisA | 5.9 | 1-(5-phosphoribosyl)-5- |
|  | hisH | 3.8 | imidazole glycerol phosphate synthase subunit HisH |
|  | pepC | 3.1 | PepC protein |
|  | glnA | 2.3 | GlnA protein |
|  | arcB | 3.0 | ornithine carbamoyltransferase |
|  | arcA | 2.3 | ArcA protein |
|  | cysK | 3.2 | O-acetylserine sulfhydrylase |
|  | metC | 4.3 | cystathionine gamma-synthase/cystathionine beta-lyase |
|  | ilvC * | 2.2 | ketol-acid reductoisomerase |
|  | ilvB * | 2.1 | acetolactate synthase catalytic subunit |
|  | gltD * | 2.1 | glutamate synthase subunit beta |
|  | hisK * | 3.9 | histidinol-phosphatase |
| Nucleotide transport and metabolism | deoD | 2.1 | DeoD protein |
|  | - | 2.1 | ribonucleoside-diphosphate reductase |
| Carbohydrate transport and metabolism | ptcC | 2.4 | cellobiose-specific PTS system IIC component |
|  | gapA | 2.4 | glyceraldehyde 3-phosphate dehydrogenase |
|  | eno | 2.6 | enolase |
|  | gpmA | 2.0 | phosphoglycerate mutase |
|  | pgi | 2.4 | glucose-6-phosphate isomerase |
|  | - | 6.1 | putative transport protein |
|  | nagA | 2.5 | NagA protein |
|  | pyk | 2.4 | pyruvate kinase |
|  | pgk | 2.3 | phosphoglycerate kinase |
| Coenzyme transport and metabolism | - | 2.9 | hypothetical protein llmg_2158 |
|  | - | 2.5 | hypothetical protein llmg_2159 |
|  | nadR | 2.2 | putative nicotinamide-nucleotide adenylyltransferase |
|  | nadE | 2.0 | NAD synthetase |
|  | ilvC * | 2.2 | ketol-acid reductoisomerase |
|  | ilvB * | 2.1 | acetolactate synthase catalytic subunit |
| Translation | gltX | 2.0 | glutamyl-tRNA synthetase |
|  | - | 4.3 | hypothetical protein llmg_1293 |
|  | serS | 2.1 | seryl-tRNA synthetase |
|  | rpmB | 2.2 | 50S ribosomal protein L28 |
|  | yfiA | 4.6 | putative sigma 54 modulation protein |
| Transcription | hrcA | 2.7 | heat-inducible transcription repressor |
|  | vacB1 | 2.2 | putative exoribonuclease R |
|  | - * | 2.4 | hypothetical protein llmg_2163 |
| Replication, recombination and repair | recA | 2.0 | recombinase A |
|  | hllA | 2.4 | HU-like DNA-binding protein |
|  | tnp712 | 2.1 | transposase for insertion sequence element IS712H |
|  | - | 4.9 | putative transposase helper protein for IS712G |
|  | matR | 2.5 | maturase |
| Cell wall/membrane biogenesis | ps356 | 4.1 | endolysin |
|  | - | 2.4 | hypothetical protein llmg_0162 |
| Posttranslational modification, protein turnover, chaperones | htrA | 2.9 | housekeeping protease |
|  | dnaK | 2.7 | molecular chaperone DnaK |
|  | clpE | 2.5 | ATP-dependent Clp protease ATP-binding subunit clpE |
|  | groEL | 2.5 | chaperonin GroEL |
|  | gcp | 2.0 | O-sialoglycoprotein endopeptidase |
|  | ahpC | 3.6 | alkyl hydroperoxide reductase subunit C |
|  | ahpF | 4.6 | alkyl hydroperoxide reductase subunit F |
|  | osmC | 2.9 | osmotically inducible protein C |
|  | clpB | 2.1 | ATP-dependent Clp protease |
| Inorganic ion transport and metabolism | sodA | 2.2 | SodA protein |
| Secondary metabolites biosynthesis, transport and catabolism | - | 2.1 | amidase |
| General function prediction only | - | 2.1 | putative NADH-flavin reductase |
|  | - | 2.9 | hypothetical protein llmg_1135 |
|  | - | 2.1 | hypothetical protein llmg_1253 |
|  | - | 2.3 | hypothetical protein llmg_0584 |
|  | hadL | 2.5 | cryptic haloacid dehalogenase 1 |
|  | - | 2.8 | aldo/keto reductase family oxidoreductase |
|  | ps350 | 3.5 | hypothetical protein llmg_0844 |
|  | gltD * | 2.1 | glutamate synthase subunit beta |
|  | hisK * | 3.9 | histidinol-phosphatase |
|  | qor * | 2.4 | quinone oxidoreductase |
| Function unknown | - | 3.1 | hypothetical protein llmg_2164 |
|  | - | 2.6 | putative 20-kDa protein |
|  | - | 3.6 | hypothetical protein llmg_1259 |
|  | - | 4.6 | hypothetical protein llmg_1260 |
|  | - | 2.2 | hypothetical protein llmg_1659 |
|  | - | 3.9 | hypothetical protein llmg_1498 |
|  | mycA | 2.2 | Myosin-crossreactive streptococcal antigen homologue |
|  | - | 3.5 | hypothetical protein llmg_1257 |
|  | - | 2.0 | hypothetical protein llmg_0152 |
|  | - | 2.5 | hypothetical protein llmg_0150 |
| Signal transduction mechanisms | - | 3.0 | hypothetical protein llmg_0093 |
|  | uspA2 | 3.7 | universal stress protein A2 |
|  | uspA | 2.4 | universal stress protein A |
|  | - | 3.2 | universal stress protein A |
|  | rcfB | 2.2 | transcriptional regulator |
|  | - * | 2.4 | hypothetical protein llmg_2163 |
| Intracellular trafficking and secretion | - | 2.5 | hypothetical protein llmg_1391 |
| No prediction | - | 2.7 | putative secreted protein |
|  | - | 3.8 | hypothetical protein llmg_1211 |
|  | - | 2.2 | hypothetical protein llmg_0317 |
|  | - | 2.1 | hypothetical protein llmg_0129 |
|  | - | 2.1 | hypothetical protein llmg_0589 |
|  | - | 8.2 | hypothetical protein llmg_0755 |
|  | - | 7.9 | putative secreted protein |
|  | - | 2.0 | hypothetical protein llmg_0757 |
|  | ps311 | 5.2 | hypothetical protein llmg_0801 |
|  | ps315 | 2.5 | hypothetical protein llmg_0805 |
|  | ps340 | 3.5 | hypothetical protein llmg_0834 |
|  | ps341 | 2.1 | hypothetical protein llmg_0835 |
|  | ps346 | 4.9 | hypothetical protein llmg_0840 |
|  | - | 2.6 | hypothetical protein llmg_1258 |
|  | - | 2.0 | hypothetical protein llmg_1385 |
|  | - | 2.4 | hypothetical protein llmg_1396 |
|  | - | 2.1 | hypothetical protein llmg_1399 |
|  | comX | 2.5 | competence regulator ComX |
|  | - | 2.4 | hypothetical protein llmg_1600 |
|  | - | 2.9 | hypothetical protein llmg_1663 |
|  | ps431 | 6.7 | hypothetical protein llmg_2111 |
|  | ps411 | 2.8 | hypothetical protein llmg_2132 |
|  | ps407 | 2.6 | hypothetical protein llmg_2136 |
|  | - | 5.1 | hypothetical protein llmg_2144 |
|  | - | 3.6 | hypothetical protein llmg_2146 |
|  | - | 3.9 | hypothetical protein llmg_2211 |
