# Supplementary material for: Metabolic and Transcriptional Analysis of Acid Stress in Lactococcus lactis, with a Focus on the Kinetics of Lactic Acid Pools
Source: PLoS One. 2013 Jul 3;8(7):e68470. doi: 10.1371/journal.pone.0068470 (PMC3700934; doi:10.1371/journal.pone.0068470)
Supplement: Table S2 — Genes with significantly lower expression profiles in L. lactis strain MG1363 suspended in KPi at pH 5.1, previously adapted to acid (grown at pH 5.1) compared with non-adapted cells (grown at 6.5). (DOC) [file pone.0068470.s007.doc]

**Table S2** Genes with significantly lower expression profiles in *L. lactis* strain MG1363 suspended in KPi at pH 5.1, previously adapted to acid (grown at pH 5.1) compared with non-adapted cells (grown at 6.5).

| **Function** | **Gene** | **Fold change** | **Description of gene product** |
| --- | --- | --- | --- |
| Amino acid transport and metabolism | dtpT | -2.6 | di-/tripeptide transporter |
|  | - | -2.3 | putative amino-acid permease |
|  | aroH | -2.7 | phospho-2-dehydro-3-deoxyheptonate aldolase |
|  | carA * | -2.6 | carbamoyl phosphate synthase small subunit |
|  | carB * | -2.6 | carbamoyl phosphate synthase large subunit |
|  | pabA * | -2.0 | p-aminobenzoate synthetase, component II |
| Nucleotide transport and metabolism | tdk | -2.2 | thymidine kinase |
|  | purC | -2.5 | phosphoribosylaminoimidazole-succinocarboxamide synthase |
|  | purS | -2.5 | phosphoribosylformylglycinamidine synthetase PurS |
|  | purQ | -2.7 | phosphoribosylformylglycinamidine synthase I |
|  | purL | -2.5 | phosphoribosylformylglycinamidine synthase II |
|  | purM | -3.7 | phosphoribosylaminoimidazole synthetase |
|  | hprT | -2.0 | HprT protein |
|  | purH | -3.1 | bifunctional phosphoribosylaminoimidazolecarboxamide formyltransferase/IMP cyclohydrolase |
|  | purD | -2.3 | phosphoribosylamine--glycine ligase |
|  | purE | -2.2 | phosphoribosylaminoimidazole carboxylase catalytic subunit |
|  | pyrB | -3.0 | aspartate carbamoyltransferase catalytic subunit |
|  | pyrDB | -6.7 | dihydroorotate dehydrogenase 1B |
|  | pyrF | -3.3 | orotidine 5'-phosphate decarboxylase |
|  | mutT | -2.7 | putative mutator protein |
|  | pyrC | -2.1 | dihydroorotase |
|  | pyrE | -3.6 | orotate phosphoribosyltransferase |
|  | pbuX | -2.3 | xanthine/uracil permease |
|  | xpt | -4.1 | xanthine phosphoribosyltransferase |
|  | carA * | -2.6 | carbamoyl phosphate synthase small subunit |
|  | carB * | -2.6 | carbamoyl phosphate synthase large subunit |
| Carbohydrate transport and metabolism | - | -2.0 | sucrose-specific PTS enzyme IIABC |
|  | - | -2.4 | hypothetical protein llmg_1358 |
|  | dexC | -2.2 | neopullulanase |
|  | dexA | -2.6 | oligo-1,6-alpha-glucosidase |
| Coenzyme transport and metabolism | hemH | -3.2 | ferrochelatase |
|  | thiD2 | -2.2 | phosphomethylpyrimidine kinase |
|  | pabA * | -2.0 | p-aminobenzoate synthetase, component II |
| Lipid transport and metabolism | accC | -2.1 | acetyl-CoA carboxylase biotin carboxylase subunit |
|  | fabD | -2.9 | malonyl CoA-acyl carrier protein transacylase |
|  | - | -3.1 | putative lysophospholipase |
|  | - | -2.8 | NAD(P)H dehydrogenase (quinone) |
|  | mvk | -2.0 | mevalonate kinase |
|  | - * | -2.4 | hypothetical protein llmg_1980 |
| Translation | hemK | -2. | putative protoporphyrinogen oxidase |
|  | truA | -2.1 | tRNA pseudouridine synthase A |
| Transcription | lytR | -3.4 | transcription regulator |
|  | rlrG | -3.0 | LysR family transcriptional regulator |
|  | relA * | -2.3 | GTP pyrophosphokinase |
|  | llrE * | -2.0 | two-component system regulator llrE |
| Replication, recombination and repair | ligA | -2.4 | NAD-dependent DNA ligase LigA |
|  | recJ | -2.2 | single strand DNA-specific exonuclease |
| Cell wall/membrane biogenesis | - | -2.1 | glycosyl transferase |
|  | mreC | -2.1 | cell shape determining protein mreC |
| Posttranslational modification, protein turnover, chaperones | trxB2 | -3.0 | TrxB2 protein |
| Inorganic ion transport and metabolism | kupB | -2.6 | putative potassium transport system protein kupB |
|  | phnD | -2.1 | phosphonate ABC transporter, phosphonate-binding protein phnD |
|  | phnC | -2.3 | phosphonates import ATP-binding protein phnC |
|  | phnB | -3.1 | phosphonate transport system permease protein phnB |
|  | - | -2.1 | cation (calcium) transporting ATPase |
|  | mntH | -2.0 | putative proton-dependent manganese transporter group C beta |
| General function prediction only | - | -2.6 | hypothetical protein llmg_2184 |
|  | - | -3.3 | hypothetical protein llmg_1988 |
|  | pbuO | -3.0 | xanthine/uracil/vitamin C permease |
|  | - | -2.0 | putative ABC transporter ATP-binding protein |
|  | maa | -2.2 | maltose O-acetyltransferase |
|  | pabC | -2.6 | putative aminodeoxychorismate lyase |
|  | - | -2.0 | hypothetical protein llmg_0332 |
|  | - * | -2.4 | hypothetical protein llmg_1980 |
| Function unknown | - | -2.3 | hypothetical protein llmg_0377 |
|  | - | -2.4 | hypothetical protein llmg_1024 |
|  | - | -2.1 | hypothetical protein llmg_1025 |
|  | - | -2.1 | hypothetical protein llmg_1760 |
|  | - | -2.1 | hypothetical protein llmg_1941 |
| Signal transduction mechanisms | pppL | -2.4 | putative phosphoprotein phosphatase |
|  | ptpL | -2.4 | protein-tyrosine phosphatase |
|  | relA * | -2.3 | GTP pyrophosphokinase |
|  | llrE * | -2.0 | two-component system regulator llrE |
| Intracellular trafficking and secretion | secY | -2.1 | preprotein translocase subunit SecY |
| No prediction | rarA | -3.5 | ArsR family transcriptional regulator |
|  | - | -2.1 | hypothetical protein llmg_1229 |
|  | - | -2.3 | hypothetical protein llmg_1230 |
|  | - | -2.6 | hypothetical protein llmg_2335 |
|  | - | -2.1 | hypothetical protein llmg_2465 |
