# Supplementary material for: Metabolic and Transcriptional Analysis of Acid Stress in Lactococcus lactis, with a Focus on the Kinetics of Lactic Acid Pools
Source: PLoS One. 2013 Jul 3;8(7):e68470. doi: 10.1371/journal.pone.0068470 (PMC3700934; doi:10.1371/journal.pone.0068470)
Supplement: Table S3 — Potassium and sodium content in perchloric extracts of L. lactis cells suspensions collected before and after glucose metabolism (40 mM). Cells were grown at pH 6.5 and suspended at pH 5.1. Values represented are averages of two experiments. (DOC) [file pone.0068470.s008.doc]

**Table S3** Potassium and sodium content in perchloric extracts of *L. lactis* cells suspensions collected before and after glucose metabolism (40 mM). Cells were grown at pH 6.5 and suspended at pH 5.1. Values represented are averages of two experiments.

|  | **Intracellular Potassium (mM)** | **Intracellular Sodium (mM)** |
| --- | --- | --- |
| Before glucose metabolism | 352.4±3.5 | 13.0±2.9 |
| After glucose metabolism | 411.3±8.7 | 109.2±13.9 |
